# COVID-19 in the homeless population: a scoping review and meta-analysis examining differences in prevalence, presentation, vaccine hesitancy and government response in the first year of the pandemic

**DOI:** 10.1186/s12879-023-08037-x

**Published:** 2023-03-14

**Authors:** Tharanika Ahillan, Matthew Emmerson, Bethan Swift, Hadiya Golamgouse, Kaiyang Song, Angela Roxas, Sakina Bano Mendha, Elena Avramović, Jatin Rastogi, Binta Sultan

**Affiliations:** 1 Barts Health NHS Trust, London, UK; 2 University of Oxford, Oxford, UK; 3 Wellcome Centre for Human Genetics, Oxford, UK; 4 University College London, London, UK; 5 King’s College London, London, UK; 6 London School of Hygiene & Tropical Medicine, London, UK; 7 Institute of Global Health, University College London, London, UK; 8 Nuffield Department of Women’s and Reproductive Health, Oxford, UK

**Keywords:** SARS-COV-2, COVID-19, Prevalence, Symptoms, Policies, Homelessness, Homeless, Scoping review

## Abstract

**Aims:**

People experiencing homelessness (PEH) have been identified as being increasingly susceptible to Coronavirus disease (COVID-19), with policies enacted to test, isolate, increase hygiene practices and prioritise vaccines among this population. Here, we conduct a scoping review of the current evidence-base pertaining to the prevalence and presentation of COVID-19 in PEH, COVID-vaccine hesitancy rates and government interventions enacted within the first year of the pandemic for PEH.

**Materials and methods:**

A systematic search was conducted on Pubmed, Cochrane, Embase and MedRxiv databases for studies reporting primary data on COVID-19 prevalence and clinical characteristics in PEH, vaccine uptake for PEH and policies enacted targeting PEH. Study qualities were assessed with The National Heart, Lung and Blood Institute’s set of Study Quality.

**Results:**

Eighty-three studies were included in our final analysis. The overall prevalence of symptomatic COVID-19 infection in PEH is estimated at 35%. The most common symptoms found were cough and shortness of breath, followed by fever. Concerns regarding vaccine hesitancy amongst PEH related to thoroughness of COVID-19 vaccine clinical trials, side effects and mistrust of the government. The main strategies implemented by governments were mass testing, adaption of healthcare service provision, provision of alternative housing, encouraging personal hygiene (hand sanitation and mask wearing), and inter-organisational communication.

**Discussion:**

In our meta-analysis, 35% of PEH with a COVID-19 infection presented symptomatically; the low prevalence of symptomatic COVID-19 infection suggests widespread testing following outbreaks would be beneficial for this group of individuals. Temporary recuperation units and measures for housing stability in the pandemic, namely provision of alternative housing and stopping evictions, were found to be highly effective. High rates of vaccine hesitancy means that education and encouragement towards vaccination would be beneficial for this vulnerable population, where comorbidities are common. Finally increased focus in research should be placed on the mental health burden of COVID-19 and the pandemic on PEH moving forwards.

**Supplementary Information:**

The online version contains supplementary material available at 10.1186/s12879-023-08037-x.

## Introduction

In December 2019, a novel beta-coronavirus known as severe acute respiratory syndrome coronavirus-2 (SARS-CoV-2) was identified in Wuhan, China; the virus likely arose zoonotically [1]. In humans, the disease is spread through direct, contact and airborne transmission [1]. Laboratory diagnosis is achieved through viral identification using reverse transcriptase-PCR (RT-PCR) of nasopharyngeal and oropharyngeal swabs, faeces and sputum [1]. The disease caused by COVID-19, was characterised as a pandemic on 11th March 2020.

Using the European Typology of Homelessness and housing exclusion (ETHOS), created by the European Federation of National Organisations working with the Homeless (FEANTSA), it is estimated over 100 million people worldwide are homeless, while over 1.5 billion live in inadequate housing [2, 3]. People experiencing homelessness (PEH) are believed to have a higher susceptibility to morbidity and mortality from infectious diseases, including SARS-CoV-2 [4]. A systematic review and meta-analysis investigating the prevalence of SARS-CoV-2 in PEH has previously been described [5]. The analysis calculated the pooled prevalence estimate of SARS-Cov-2 in PEH to be 31.59% (95% CI, 2.48–42.71%) in the context of outbreak situations, and 2.32% (95% CI, 1.30–3.34%) in non-outbreak situations.

Transmission risks in homeless accommodation settings and barriers to preventive behaviours, such as social distancing and regular handwashing, may place PEH under higher vulnerability to COVID-19 infection. In addition, PEH experience excess mortality due to cardiovascular and chronic respiratory diseases, diseases which increase the risk of severe COVID-19 [6]. Finally, different rates of mental health conditions (for example depression and schizophrenia) in PEH compared to the general population may affect their ability to adapt to measures put in place to prevent the spread of infection during the pandemic [7]. These factors may lead to altered risk and outcomes of COVID infection in PEH compared to the general population.

A variety of policies have been enacted by governments to reduce COVID-19 transmission rates [8], particularly amongst PEH. However, the effectiveness of these policies have yet to be fully established.

In particular, a key tool has been the use of vaccines, which protect against COVID-19. However, vaccine hesitancy has been cited as a barrier to the uptake of vaccines once they are available [9]. Studies have attempted to describe factors associated with vaccine hesitancy within the general population [10, 11]; as of yet, no study has summarised this barrier in PEH.

Given the increasing list of COVID-19 variants and the evolving nature of governments’ policies to tackle the pandemic, we have decided to focus on the first year of the pandemic.

As such, this scoping review aims to collate evidence relating to the following questions regarding COVID-19 in PEH:

1. What is known about how COVID-19 manifests in patients defined as homeless in the first year of the pandemic:
  - Are patients defined as homeless at a higher risk of developing symptomatic COVID-19?
  - Do patients defined as homeless present differently to the general population, with regards to COVID-19?
2. What is known about the rates of COVID-19 vaccine hesitancy amongst PEH in the first year of the pandemic?
3. What is the evidence on the effects of government policies and strategies (synonymous in our review with non-pharmaceutical interventions) brought in to specifically address PEH during the first year of the COVID-19 pandemic?

To the best of our knowledge, this is the first scoping review examining COVID-19 vaccine hesitancy within the homeless population compared to the general population, thus providing a vital addition to the evidence base.

## Materials and methods

### Protocol

Preferred Reporting Items for Systematic Reviews and Meta-Analyses (PRISMA) guidelines were followed and the study protocol was prospectively registered on PROSPERO (CRD42021262166) [12].

### Inclusion criteria

English language reports of all study types (including quantitative, qualitative and mixed methods research) presenting or reporting primary clinical data were examined. Studies focussing on the prevalence and clinical characteristics of COVID-19 in patients defined as homeless, reviewing COVID-vaccine uptake or analysing government response policies and strategies amongst the homeless population were eligible for inclusion. Homelessness was defined according to the FEANTSA-ETHOS definition [3]. Studies conducting original analyses on previously reported data were also included. The rationale behind keeping a broad inclusion criteria was to provide the most accurate snapshot of the research field in view of evolving literature during the first year of the pandemic.

Both peer-reviewed and pre-print (non peer-reviewed) literature were included to account for the rapid evolution of the evidence-base during the current crisis, and to minimise the impact of publication bias [13]. Where applicable, only RT-PCR-confirmed SARS-CoV-2 infections were considered to constitute as COVID-19 infection. Patients of any age, gender, nationality and healthcare setting were eligible for inclusion.

### Exclusion Criteria

Exclusion criteria were: (1) papers which did not fit the inclusion criteria, (2) protocol-only publications, (3) commentaries or opinion pieces not presenting any primary data*,* (4) systematic reviews not presenting any original analyses, (5) clinical trials with no results published.

### Sources and search strategy

Searches were conducted in PubMed, Embase (OVID), MedRxiv and within grey literature databases for studies published between 1/12/2019 and 20/06/2021 (Additional file 1: Appendix A; Additional file 2: Appendix B). The search strategy was created by TA and ME, then independently verified by HG, KS, AR, EA and JR. Results were imported into Zotero to remove duplicates.

### Study selection

Study selection followed methodology set out by previous systematic reviews ([5, 14]). De-duplicated results were distributed across three reviewer pairs; titles and abstracts were independently screened for eligibility by two reviewers via Rayyan [15]**.** Disagreements were resolved by an independent third reviewer. Full texts of included papers were primarily independently screened by two reviewers with disagreements resolved by an independent third reviewer. A secondary independent screening of full texts of included papers by two reviewers took place; disagreements were resolved as before.

### Data collection and quality assessment

A standardised data extraction tool was developed by TA and ME and piloted by six reviewers on two randomly chosen papers each. The final included papers underwent single-reviewer data extraction. The National Heart, Lung and Blood Institute’s (NHLBI) set of Study Quality Assessment tools [16] were used to assess the quality of included papers; a standardised scoring system was created for each criteria in the Study Quality Assessment Tools. All quality appraisals were checked by two reviewers and converted into ‘Poor’, ‘Fair’ and ‘Good’ ratings through consensus between two reviewers; no papers were excluded on the basis of their quality ratings.

### Synthesis of results

All papers were reviewed for relevance to the primary questions of (i) risk, (ii) clinical presentation, (iii) vaccine hesitancy and (iv) government policy and strategy. A descriptive summary was conducted for each question, with quantitative synthesis conducted where possible.

### Statistical analysis

With respect to clinical characteristics, we extracted relevant parameters from quantitative studies to calculate a pooled prevalence rate for symptomatic COVID-19 cases. We assumed that the prevalence of symptomatic individuals was randomly sampled from a distribution and therefore applied a random-effects method with a Freeman-Tukey Double Arcsine Transformation to stabilise the variances [17]. We calculated I^2^ to assess the proportion of variance due to heterogeneity. We produced funnel plots and regression based statistical testing by Egger et al. to assess risk of publication bias [18]. The analysis was performed using Stata version 17 with commands metaprop and meta.

### Descriptive analysis

For our analysis on specific symptoms of COVID-19 in PEH, we constricted our analysis to descriptive measures of the prevalence of each sign/symptom in each paper. For our analysis on vaccine hesitancy and non-pharmaceutical interventions, we performed a descriptive summary only. This was decided upon the paucity of evidence for vaccine hesitancy, and the design of the papers available for policy analysis.

## Results

### Study selection

**Fig. 1:**
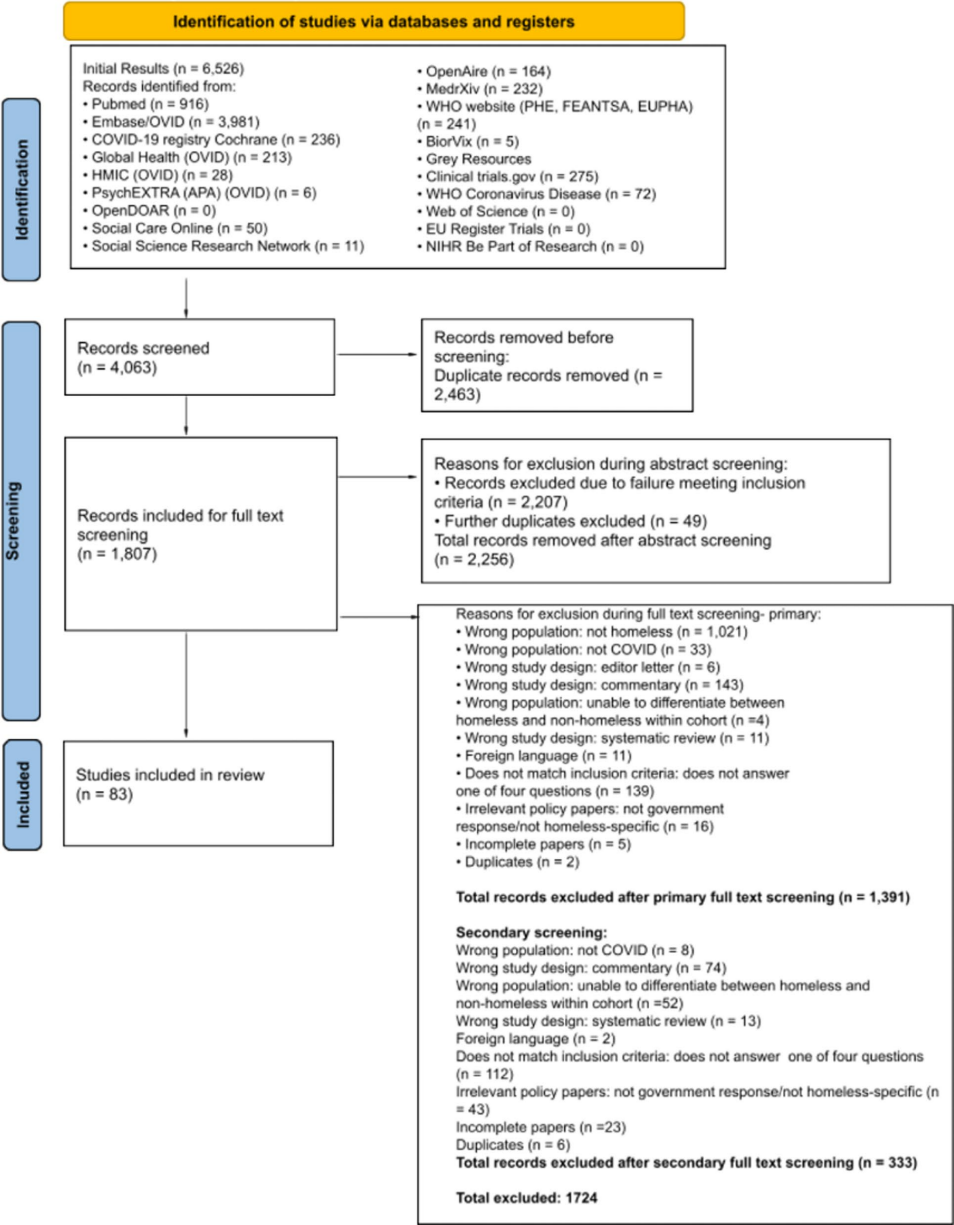
Flowchart of included and excluded studies (PRISMA diagram). 6 case reports/series, 18 observational studies (4 cohort studies and 14 cross-sectional studies), 4 commentaries, 5 modelling studies, 34 qualitative studies, 15 reports and 1 rapid review were included

Eighty-three studies were included in the final synthesis as shown in the PRISMA diagram (Fig. 1): 16 studies were used to analyse clinical characteristics of COVID-19 positive PEH, 7 studies were used to calculate vaccine hesitancy rates, 62 studies were analysed with respect to government interventions pertinent to COVID-19 amongst the homeless population and a further 2 studies identified in order to provide an update on prevalence amongst the homeless population.

### Study characteristics

Characteristics of the study populations are depicted in Table 1 and Additional file 4: Appendix D and Additional file 5: Appendix E. Most of these studies took place in the US (n = 38, 45.8%) and UK (n = 21, 25.0%). Regarding homelessness status, accounting for overlap between studies, 35 studies looked solely at houseless PEH, 6 solely at roofless PEH, with 1 examining PEH classed as insecure and inadequate respectively. 17 studies looked at houseless and roofless PEH, with 16 studies looking at other combinations; 9 studies focused on hospitalisations or were otherwise unclear. Mean age for clinical characteristics was 53; for vaccine hesitancy mean age was 48.2. All papers in which sex was given reported a higher percentage of males included in their analysis compared to females. When conducting quality analysis for risk of bias, 10 papers were assessed as poor, 61 papers were assessed as fair, and 12 papers were assessed as good quality.

**Table 1:** Characteristics of studies describing clinical characteristics

Author, year	City (Country), study period	Total sample size*	Population (FEANTSA definition)	When/how symptoms were assessed	Age +	Male sex, n (%)
Fields et al. (2021)	Salt Lake, (USA), March–May 2020	127	Shelters, unsheltered and encampment (houseless, roofless)	Obtained during contact tracing during interview or obtained by a shelter worker on the investigation form	Median = 48 (range = 4–89)^^^	159 (94.1%)^^^
Samuels et al. (2020)	Rhode Island, (USA), April 2020	35	Shelter only (houseless)	Reported by participants at the time of testing	18–39 = 9 (25%), 40–64 = 23 (66%), > 65 = 3 (9%)	26 (74%)
Imbert et al. (2020)	San Francisco (USA), March 29-April 11 2020	100	Shelter only (houseless)	Extracted from case interviews and isolation and quarantine hotel referral forms	Median = 54 years (range = 22–77)^	215 (84%)^
Kiran et al. (2021)	Toronto (Canada), April 1–July 31 2020	44	Shelter only (houseless)	During mobile outreach by interview. Only occurred when sufficient staff were available for outreach	Mean = 48.3 (sd = 18.0)^^^	72 (92%)^^^
Cha et al. (2021)	Several states (USA), 1 March-31 May, 2020	199	Hospitalizations only (unclear)	Upon admission	Median = 53 (range = 48–58)	165 (83.5% weighted)
Redditt et al. (2020)	Toronto (Canada), April 2020	24	Shelter only (houseless)	Any time during 14 days posttesting	Mean age = 38.7 (sd = 11.0)^^^	22 (88%)^^^
Baggett et al. (2020)	Boston (USA), 2 April-3 April, 2020	147	Shelter only (houseless)	Reported by participants at the time of testing	Mean = 53.1 (sd = 12.8)	124 (84%)
Ghinai et al. (2020)	Chicago, (USA), March 1-May 1 2020	406	Shelter only (houseless)	At date of specimen collection and in the following 2 weeks	< 40 years = 80 (18.8%), 40–55 years = 32 (31.0%) and > 55 years = 214 (50.2%)^^^	292 (69.5%)^^^
McCormick et al. (2020)	Denver, (USA), May–July 2020	61	Shelters, unsheltered and encampment (houseless, roofless)	Reported by participants at the time of testing	Median = 47 (range = 36–56)	59 (97%)
Roland et al. (2021)	Brussels (Belgium), April 27–June 10, 2020	89	Shelter only (houseless)	Reported by participants at the time of testing	< 40 years = 49 (62%) ≥ 40 years = 30 (38%)^^^	62 (68.1%)^^^
Gaeta et al. (2020)	Boston, USA	1	Shelter, followed by hospitalisation (houseless)	Reported by participants at the time of testing	N/A	Male- 100%
Tobolowsky et al. (2020)	Washington, USA	195 (shelter residents)	Shelter (houseless)	Symptom screening occurred after SARS-CoV-2 testing via 14 symptom screening events	The median age was 61 yeats (range = 50–73 years)	187 of 195 (96%) residents tested were men
Ghalamkarpour et al. (2020)	N/A	1	Hospitalisation, from shelter (houseless)	During admission to hospital	45	100%
Przydizial et al. (2020)	N/A	1	Hospitalisation from shelter (houseless)	During admission to hospital	51	100%
Byrnes et al. (2020)	N/A	2	Hospitalised (unclear)	Presented and was tested	36	100%
Prodanuk et al. (2021)	Canada	1	Hospitalised (houseless)	Presented and was tested	16	0%

*Sd* standard deviation

*Includes those positive with COVID-19 where symptom information was available

^+^Information on age was not always available for only the positive COVID-19 cases with symptom information

^^^Age/sex information given in aggregated form

### Narrative synthesis

#### Prevalence of COVID-19 in homeless patients

We identified two quantitative cross sectional studies relating to COVID-19 and homelessness that met the inclusion criteria that were not included in the previous systematic review looking at homelessness and COVID-19 [5]. The first [19] took place in Brussels, Belgium and involved testing 1,994 adults in 52 shelters between April and June 2020. Overall prevalence was estimated at 4.6% over this time-period. The second [20] analysed population-based surveillance data of COVID-19-associated hospitalisations between 1 March and 31 May 2020 in the United States of America. The study found 201 (2%; 95% CI, 1.5%-2.4%) of people hospitalised with COVID-19 had a housing status of “homeless”.

#### Prevalence of symptomatic SARS-CoV-2 infection

Overall, the 16 studies [19–34] reporting on presentation in COVID-19 positive homeless patients described 1433 adults with laboratory confirmed COVID-19 from 3 different countries.

**Fig. 2:**
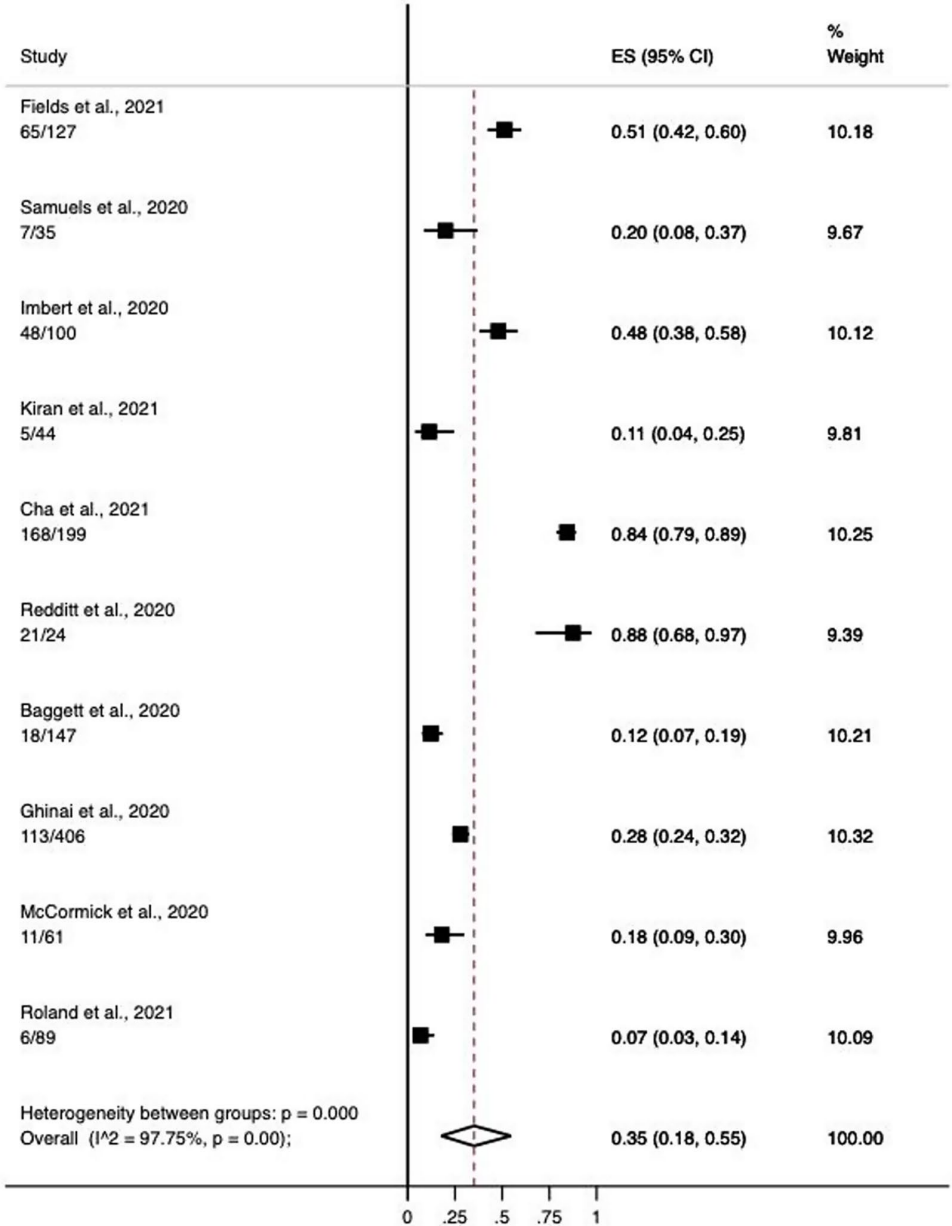
Proportion of hospitalized homeless COVID-19 patients. The pooled prevalence of COVID-19 in the homeless population across 10 cohort studies was 35% (95% CI 18–55%)

10 studies were utilised to calculate the pooled prevalence of symptomatic SARS-CoV-2 infection (Table 1). We pooled the measures in a random-effects meta-analysis model to adjust for the varying settings and contexts (n = 1,232). The pooled prevalence rate of symptomatic PEH was 35% (95% Confidence Interval, 95% CI 18–55%). There was significant heterogeneity in the estimates for each group (p < 0.001) with I^2^ = 97.75% (Fig. 2).

Funnel plots for the studies included in this meta-analysis are presented in the supplementary material (Additional file 3: Appendix C). Results from the Egger test suggest absence of significant publication bias (*p* = 0.86).

#### Clinical characteristics of SARS-CoV-2 infection in homeless individuals

We included 6 observational studies in this analysis [20, 24, 29–31, 34] (Table 1). All reported prevalence of fever, cough and shortness of breath, which ranged from 2% (3/147) [30] to 51% (102/199) [20], 6% (2/35) [34] to 63% (56/89) [31] and 0% (0/35) [34] to 45% (89/199) [20], respectively. Three studies [24, 29, 34] reported on prevalence of loss of smell/taste, which ranged from 6% (2/35) to 29% (7/24) and 4 studies [24, 29–31] reported runny nose as a symptom at a prevalence ranging from 1% (2/147) to 25% (6/24). Two studies [24, 29] reported sore throat with prevalence ranging from 22% (14/65) to 25% (6/24) and the same two studies reported headache prevalence as 20% (13/65) to 33% (8/24)) and chills as 13% (3/24) to 26% (17/65). Three studies [24, 29, 34] reported on myalgia 6% (2/35) to 29% (19/65) and one study [29] reported chest pain (17%, 4/24) and dizziness (17%, 4/24) as symptoms. No studies reported on combination of symptoms and no studies stratified symptoms by age.

We also included 6 case reports in this analysis, with a total of 7 patients ([21–23, 26, 27, 33]. Within the case reports, likewise the most common symptoms found were cough (n = 4, 57%) and shortness of breath (n = 4, 57%) as seen in Table 2. No study reported loss in sense of smell or taste in the patients. 3 patients in the case reports were found to be hypoxic on admission. 3 case studies discussed the laboratory and radiological findings on admission [23, 26, 27]. Laboratory findings included non-specific rises in inflammatory markers [23, 27] and point of care tests, such as elevated postprandial glucose level [21]. In two cases imaging was carried out [26, 27]; this found changes on CXR and CT scan [26] but not on initial presentation in the other case [27].

**Table 2:** Clinical presentation of COVID-19 in homeless patients within case series and case reports

Features	Gaeta et al. [16] (n = 1)	Tobolowsky, [17] (n = 1)	Ghalamkarpour et al. [18] (n = 1)	Przydzial et al. [21] (n = 2) *	Byrnes et al. [22] (n = 1) *	Prodanuk et al. [28] (n = 1)
Cough	n = 1	n = 1		n = 1		n = 1
Shortness of breath (SOB)		n = 1		n = 2		n = 1
Fever		n = 1	n = 1		n = 1 Presented two days later	
Loss of smell and/or taste						
Runny nose/rhinorrhoea	n = 1	n = 1				
Nausea and/or vomiting				n = 2	n = 1	
Diarrhoea					n = 1	
Muscle aches/myalgia	n = 1			n = 2		
Sore throat	n = 1	n = 1				
Recurrence of erythroderma			n = 1			
Slurred speech					n = 1	
Pinpoint pupils					n = 1	
Acute encephalopathy		n = 1			n = 1	
Chest pain						n = 1
Hypoxia/low SpO_2_				n = 2	n = 1	
Elevated glucose	n = 1					
Normal White Cell Count (WCC)			n = 1	n = 2	n = 1	
Normal lymphocytes				n = 1		
Raised lymphocytes				n = 1		
Raised neutrophils			n = 1			
Normal lymphocytes			n = 1			
Low lymphocytes					n = 1	
Low haemoglobin (Hb)			n = 1			
Normal Haemoglobin (Hb)				n = 2		
Low platelets				n = 1		
Low serum albumin			n = 1			
Raised erythrocyte sedimentation rate (ESR)			n = 1	n = 1	n = 1	
Raised C-Reactive Protein (CRP)			n = 1		n = 1	
Normal lactate dehydrogenase (LDH)			n = 1			
Radiological			n = 1 Computed Tomography Scan (CT): normal	n = 1 (Chest X-Ray: bilateral infiltrates. Chest Computed Tomography Scan (CT): ground glass opacities)	n = 1 Computed Tomography Scan Head (CT Head): no acute infarct or intracranial haemorrhage Chest X-Ray (CXR): normal Magnetic Resonance Imaging (MRI): multiple focal enhancing lesions	

*Described as ‘homeless’ within case report/case series

n = 1 represent 1 patient experiencing this symptom/investigation result

n = 2 represents 2 patients experiencing this symptom/investigation result

### COVID-19 vaccine hesitancy in the homeless population

Analysis of vaccine hesitancy rates amongst the homeless population has predominantly occurred across Europe and North America with figures ranging from 35.7 to 48% [35, 36].

Several studies highlighted the common concerns of PEH regarding the COVID-19 vaccine. A cohort study involving 94 PEH in Oakland and San Francisco highlighted suspicion about adverse side-effects, such as the belief that a sore arm post-vaccine is an example of vaccine-induced disease, and doubts over the robustness of the COVID-19 vaccine clinical trials [37]. These views were echoed in another survey conducted in the UK [38]. These factors also underpinned reluctance to engage in COVID-19 vaccine trials.

In the Democratic Republic of Congo, questionnaires distributed to people living in internally displaced persons camps [39] revealed that having a COVID-19 vaccine was not a high priority due to longstanding issues in the cleanliness and safety of their living conditions. People did not see the importance in being vaccinated when their living conditions were so unsanitary. Elsewhere, in the UK, qualitative interviews of 32 migrants, refugees and asylum seekers highlighted that the language barrier and a lack of understanding of entitlement to the vaccine underpinned hesitancy. Out of 10 individuals asked, all thought that their immigration status would be checked upon receiving a vaccine [40].

To date, the largest study addressing vaccine hesitancy in the homeless population was conducted in France: 235 individuals across Paris and Lyon were questioned about the COVID-19 vaccine [41]. The rate of vaccine hesitancy (40.9%) was in line with the general population and mirrors findings from a separate study based in Vatican City State where 35.7% of homeless individuals expressed hesitancy [36]. Longchamps et al. [41] highlighted a significant association between low intention to be vaccinated against COVID-19 and low health literacy, being a woman, and being a legal resident in France. Moreover, a survey of 90 PEH in Los Angeles [35] discovered an association between vaccine hesitancy and obtaining COVID-19 related information from sources other than official health bodies, including social media, friends and family.

### Policies addressing COVID-19 in the homeless population

Several non-pharmaceutical interventions (NPIs) were enforced by governments pertaining to PEH in response to COVID-19. The 5 groups of NPIs identified in this analysis were mass testing, adaptation of healthcare service provision, providing alternative housing, encouraging personal hygiene (hand sanitation and mask wearing) and inter-organisational communication.

#### Mass testing

Mass testing (both PCR and lateral flow) of symptomatic and asymptomatic individuals following identification of a positive COVID-19 case was described as a feasible, successful and highly effective strategy. Papers described these taking place in a number of locations including Boston [21, 42, 43], Montpellier [44], Berlin [45], Toronto [28]. Conversely, attempts at mass testing homeless shelters without an outbreak present had varying outcomes. Studies based in Detroit [46], San Diego [47], California [48] and Michigan [49] reported success while attempts in Boston [50] and Berlin [45] were deemed not feasible due to lack of resources.

#### Adaptation of healthcare service provision

The three main strategies to combat COVID-19 in PEH were the creation of temporary recuperation units, implementation of street outreach teams and introduction of tele-health and remote consultations. The majority of papers describing temporary recuperation units placed these in Boston. The COVID Recuperation Unit [51–53] helped PEH recover from COVID while providing concurrent aid for mental health and substance addition needs; the unit helped decrease PEH hospital admissions by 28% [51]. The Boston Hope Field Hospital [54] focused on addressing the major mental health needs and was reported as beneficial for PEH. Another medicalised hotel in Madrid [55] found lack of quarantine housing conditions was a key reason for admission; hence the foundation of a medicalised hotel proved to be useful. Street outreach of healthcare was implemented and well received in both the UK [56] and US [57] by PEH.

Mixed feedback was given of current services which were modified to telehealth services. Some homeless residents felt valued and supported [58], new people were found who had not sought support before [59, 60] and hospital admissions were reduced [61]. Likewise positive feedback was seen in response to GP surgery adaptations including remote registration systems to enable PEH to register and attend healthcare appointments [62, 63]. However, homeless populations seemed to be using tele-health services less than the non-homeless population [64]; lack of technology access was reported as one issue [65], while others found it difficult to access mental health support [66] or discuss mental health concerns over the phone [65], thus reducing patient-doctor rapport [62, 63, 67].

#### Alternative housing

The ‘Everyone In’ campaign in England was the most referenced alternative housing scheme. A number of papers talked about its success in providing temporary accommodation for ‘over 33,000 people by November [2020]’ and over 90% of the homeless population being offered accommodation within the first month and a half’ [68–70]. Some papers which surveyed the rehoused homeless population found this had increased physical health, engagement with other health services such as the addiction service [71] and possibly helped to lower infections and deaths [72]. This was echoed in a similar scheme in South Africa [73].

However, the unavailability of accommodation to some of the population, especially non-UK nationals [74, 75], logistical issues leading to unpredictability and adverse effects on mental wellbeing [76, 77], lack of social distancing with positive cases [78] and a lack of support for the young homeless population [79, 80] were also raised. In the latter case, one youth homeless charity still experienced a 33% increase in calls during the pandemic [80]. This was referenced amongst other policies in the FEANTSA report on rough sleepers [81] which stressed that there was different and/or decreased dissemination of information to migrants. One study found that the concordant ban on evictions in the UK [80], which in other papers had been lauded, did not prevent an increase of 33% in calls to Centrepoint [helpline].

Other locations implemented similar rehousing schemes such as Singapore [82], Scotland [83], Dallas [84] and Arlington County [85] all of which were taken up by the homeless populations. However, a survey in a migrant worker sheltered home in India found high anxiety about being far from their family and lack of psychological support [86]. Other regions, such as San Francisco [87, 88] and Austin [89] implemented isolation and quarantine accommodation to give the homeless population shelter for the duration of their isolation. Over 80% of inhabitants finished their stay and only 4% of inhabitants required hospital admission, representing a significant alleviation of hospital facilities. This evidence was compounded with a number of modelling studies [6, 43, 90, 91] which found that provision of non-hospital non-congregate accommodation was a highly effective measure.

#### Personal hygiene

Personal hygiene measures focused on hand washing [92, 93] and mask wearing [94]. Lack of public facilities due to public services closing during the pandemic was found to be a large barrier to maintaining personal hygiene [93]. Implementation of mobile, easily formed hand washing stations was received well by the homeless population [92]. Mandated mask wearing in shelters was made easier through allowing personalised cloth masks to be worn rather than surgical masks [94]. Two studies [73, 95] described policies to prevent cross contamination by the supply of disposable cups [95] and opioid-substitution therapy [96] for drug users, both citing success in empowerment of individuals in reducing risk of COVID-19 transmission and implementation as part of a larger harm reduction and community engagement strategy.

#### Inter-organisational communication

The importance of governments and non-governmental organisations (NGOs) working in tandem were highlighted in Iran [97]; conversely in Brazil, failure to do so meant NGOs could become overwhelmed [98]. Furthermore papers described how a lack of communication between local and national governments had led to confusion over vaccine prioritisation [99] and outbreak reporting [100] in the homeless population, leading to less efficient distribution of resources for PEH.

## Discussion

We included results from 83 studies across 15 countries.

### Clinical characteristics

We estimated the overall prevalence of symptomatic COVID-19 infection in PEH at 35%. The most common symptoms in the observational studies and case studies were cough and shortness of breath, with fever most common in the observational studies but not within the case reports. This was similar to a previous systematic review on the general population of COVID-19 patients [101], which found that fever, cough, fatigue, and dyspnoea were the most commonly reported clinical features of coronavirus disease.

The CDC estimates that 35% of all COVID-19 cases are asymptomatic, with 65% as symptomatic. This is much higher than our estimate of symptomatic COVID-19 in the homeless population (35%) and suggests that homeless people are less likely to be symptomatic; this implies that screening based on symptomatic COVID-19 is not adequate in capturing the true prevalence of COVID-19 in the homeless population. Given that PEH are at a higher risk of comorbidities, such as cardiovascular disease and chronic infections [102], poorer outcomes may be evident upon COVID-19 infection in PEH. Additionally, PEH have had high numbers of hospitalisations during influenza pandemics, suggesting they are particularly susceptible to viral diseases [103]. Strategies to quickly identify COVID-19 cases in PEH, must take the above into account.

### Vaccine hesitancy

The seven studies on vaccine hesitancy found that many of the homeless population’s concerns regarding thoroughness of COVID-19 vaccine clinical trials, side effects and mistrust of the government are in-line with those of the general population [104].

The homeless population generally has a higher rate of vaccine hesitancy of 35.7–48% as described in two studies [35, 36] compared to the general population which is estimated to range from 22.4 to 35% [105–107]. However, all this information has come from Europe and North America. This discrepancy may be elucidated by the increased tendency of the homeless population to rely on social media as the leading source of health-related information [41]. There has been widespread vaccine-sceptic/anti-vax information circulated through these mediums [108]. This, in conjunction with lower health-literacy rates [109] may underpin certain beliefs of this population [37].

On the whole, it is vital that the vaccine uptake rates within the homeless population are maximised given that this group is especially vulnerable to developing morbidity and mortality from COVID-19 infection and will aid in health equity [4].

### Policies

The strategies and policies implemented by governments for the homeless population in response to COVID-19 were within the following themes: mass testing, adaptation of healthcare service provision, provision of alternative housing, encouraging personal hygiene (such as hand sanitation and mask wearing) and inter-organisational communication.

The high incidence of COVID-19 positive cases found in response to outbreaks within shelters, coupled with the fact that these individuals did not significantly display any more clinical features (cough, fever) compared to non-COVID infected individuals [42] further suggests the need for a universal testing strategy implementation amongst the homeless population in shelters in outbreak situations. However, given the low yield for asymptomatic screening as a preventative measure and limited laboratory capacity and manpower [44], universal daily testing may not be as effective. Instead daily symptom screening in homeless shelters may be considered as an alternative [49].

In addition, the studies showed that high risk homeless shelters with high community incidence are more prone to outbreaks even with intensive infection control practices. This reflects the necessity of non-congregate housing for the homeless. Indeed, temporary recuperation units were found to be highly effective in diverting cases that would otherwise have resulted in hospitalisation for isolation and quarantine, thereby reducing the strain on hospital inpatient capacity [43, 51, 88, 90].

This adds strength to the evidence base suggesting that provision of alternative care sites for COVID-19 management, alongside daily symptom screening and PCR testing of individuals with positive screening results is the most efficient and cost-saving strategy amongst the homeless population [41]. However, the reality as evidenced by the studies analysing “Everyone In” suggests that this governmental intervention should ensure that logistical capabilities, such as having enough space within accommodation social distancing, and providing more certainty of availability of accommodation for PEH, should be considered for successful implementation. This can be aided by effective restructuring of healthcare services such as GP surgeries to ensure PEH are able to access medical support more easily [62, 63] and better communication between governments and NGOs [97].

## Limitations/methodological considerations

We included both major databases, grey literature, governmental and non-governmental associations, and charities’ websites. All steps of the review have been conducted by two reviewers to ensure quality assurance.

Our review also has some limitations which need to be considered.

Firstly, the majority of included papers are from the USA and as we only used English search terms, hence it is possible that not all relevant papers were captured or included in our study. Therefore, our findings may not be generalisable to other countries or settings as transmission may differ depending on cultural or social setting. It has been found previously that resource-poor governments are unable to respond efficiently in a pandemic during natural disasters which lead to increased social inequities [110]. Thus, it is important to expand the evidence base to lower- and middle-income countries, to ensure the policies implemented are representative of the global population affected by COVID-19.

Secondly, prevalence of different COVID-19 symptoms was often not the primary aim of the included studies, limiting our analysis. Often symptomatic homeless patients with COVID-19 were largely described coincidentally in cohort studies, where the primary objective was not to investigate the clinical presentation. Consequently, relevant confounders, including demographic and social factors and medical comorbidities were often not considered or reported.

In addition, where demographic factors were reported, the majority of the population were of male sex. This limits the inference that can be made, given that these findings may not be generalisable to homeless females. Similarly, many of the studies looked at PEH in shelters, with a paucity of evidence amongst other PEH groups including those in insecure housing, and rough sleepers, making it difficult to extrapolate our findings to the homeless population at large.

With regards to vaccine hesitancy, many studies examining vaccine hesitancy within the homeless population have not utilised a non-homeless population as a control group. Some studies assess vaccine hesitancy based on the offer of an actual vaccine whilst others combine this with data based on a hypothetical vaccine. This is an important distinction to make, as vaccine hesitancy towards a hypothetical vaccine is not necessarily equivalent to hesitancy towards an actual vaccine. Therefore, it is difficult to elucidate whether the homeless population express greater reluctance towards the vaccine than the general population, and if so, whether this is associated with particular social, demographic or other factors.

Finally, because of our restricted search timeframe, there may be papers published after our search date which contain information about the prevalence, clinical characteristics, vaccine hesitancy rates and policy outcomes from the first year of the pandemic which have not been included in this paper. Thus, the conclusion described in this paper may be different to those which can be inferred from data about the subsequent years of the pandemic. As such, an updated search of this paper to examine these factors in PEH in subsequent years of the pandemic would be beneficial.

## Implications for practice, policies and future research

We have identified important points that should be addressed by future research.

### Future research

1. There is a need for separation amongst different groups related to homelessness. For instance, in the case of symptoms related to COVID-19, staff working with PEH, and PEH themselves were not considered in the research as two separate groups. Whilst addressing the staff population who work with the homeless population can mitigate transmission of SARS-CoV-2 infection amongst the homeless population, the paucity of evidence means that the evidence base would benefit from this separation when studies are carried out. Similarly, within the policy papers, there is a need to differentiate between the different types of homelessness, including rough sleepers and sheltered individuals, to ascertain a more nuanced understanding of clinical characteristics and vaccine hesitancy between the various groups and ensure measures implemented target the entire PEH population.
2. Additionally, there is a need to provide demographic information pertaining to clinical characteristics and vaccine hesitancy in particular, as often this information is given for the homeless population at large but not those specifically who have COVID-19.
3. With the current speed in which research is being published in this field, it is important that there is more global representation particularly in relation to encouragement and funding for research to come from outside Europe and North America.
4. Finally, from qualitative evidence, there is evidence suggesting temporary accommodation can relieve anxieties within PEH; more research regarding the mental health implications of the COVID-19 pandemic on the homeless population is required to help guide policies to address this moving forwards in this pandemic.

### Future practice and policies

1. With relation to vaccine hesitancy, there is a greater need to deliver and tailor COVID-19 vaccine information to the homeless population; if this is fulfilled, this can reduce the reliance of this population on social media for information.
2. Success has been found in using community healthcare outreach programmes, such as existing staff at shelters or at food banks, in delivering policies during the pandemic, and this can continue moving forward in delivering the vaccine to the homeless population. The role of using this existing infrastructure should not be underestimated in this process and has the potential to address the barriers of infrastructure, transport, mistrust and outreach to the homeless population to allow access to COVID-19 vaccine sites.
3. We encourage the use of mass testing as a response to outbreaks, with symptomatic screening done daily within shelters in non-outbreak situations. Implementation of isolation and quarantine accommodation are highly effective in avoiding hospital admissions.

The authors stress that many of the recommendations for future practice and policies are not different from previous recommendations to increase uptake of healthcare initiatives within the homeless population; the effectiveness of creating housing stability and capitalising on existing policies put in place to do so is integral to improving COVID-19 prevalence, clinical characteristics, and outcomes within this population, and increasing uptake of COVID-19 vaccines as we move forward.

## Conclusions

This review has estimated the prevalence of symptomatic COVID-19 infection in PEH, described the clinical profile of these individuals and explored the reasons behind vaccine hesitancy and the public health policies that have been implemented to protect this high-risk group, focusing on the first year of the pandemic. Policies have included: mass testing, adaptation of healthcare service provision, provision of alternative housing, stopping evictions, encouraging personal hygiene (such as hand sanitation and mask wearing) and inter-organisational communication.

In our meta-analysis, 35% of PEH with a COVID-19 infection presented symptomatically, suggesting routine and regular testing would be beneficial for this group of individuals. The low prevalence of symptomatic COVID-19 infection, combined with high rates of vaccine hesitancy, means that frequent testing as well as education and encouragement to be vaccinated, would be highly beneficial for this vulnerable population, where comorbidities are common. Finally, increased focus should be placed on the mental health burden of COVID-19 and the pandemic on the homeless population moving forwards.

## Supplementary Information

**Additional file 1. **Table of search terms.

**Additional file 2. **List of databases searched.

**Additional file 3. **Funnel plots for meta-analysis.

**Additional file 4.** Characteristics of studies describing vaccine hesitancy.

**Additional file 5.** Characteristics of studies describing policies (non-pharmaceutical interventions).

## Data Availability

The results, data and figures in this manuscript have not been published elsewhere, nor are they under consideration by another publisher. All the material is owned by the authors and/or no permissions are required. The dataset used and analysed for included studies during this study are available from the corresponding author on reasonable request.

## Abbreviations

COVID-19: Coronavirus-disease-2019
CT: Computer tomography
PEH: Persons experiencing homelessness
FEANTSA: European Federation of National Organisations working with the Homeless
ETHOS: European Typology of Homelessness and housing exclusion

## References

[1] Umakanthan S, Sahu P, Ranade AV, Bukelo MM, Rao JS, Abrahao-Machado LF (2020). Origin, transmission, diagnosis and management of coronavirus disease 2019 (COVID-19). Postgrad Med J.

[2] Global Homelessness Statistics [Internet]. Homeless World Cup. https://homelessworldcup.org/homelessness-statistics/

[3] Edgar B, Harrison M, Watson P, Busch-Geertsema V. Measurement of Homelessness at European Union Level [Internet]. European Commission: Employment, Social Affairs and Equal Opportunities DG; 2007. https://ec.europa.eu/employment_social/social_inclusion/docs/2007/study_homelessness_en.pdf

[4] Leifheit KM, Chaisson LH, Medina JA, Wahbi RN, Shover CL (2021). Elevated mortality among people experiencing homelessness with COVID-19. Open Forum Infect Dis.

[5] Mohsenpour A, Bozorgmehr K, Rohleder S, Stratil J, Costa D. SARS-Cov-2 prevalence, transmission, health-related outcomes and control strategies in homeless shelters: Systematic review and meta-analysis. eClinicalMedicine [Internet]. 2021 Aug 1 [cited 2022 May 30];38. https://www.thelancet.com/journals/eclinm/article/PIIS2589-5370(21)00312-6/fulltext 10.1016/j.eclinm.2021.101032 PMC8298932 34316550

[6] Lewer D, Braithwaite I, Bullock M, Eyre MT, White PJ, Aldridge RW (2020). COVID-19 among people experiencing homelessness in England: a modelling study. Lancet Respir Med.

[7] Sharma M, Aggarwal S (2020). Homeless persons with mental illness during COVID-19. Asian J Psychiatry.

[8] Umakanthan S, Bukelo MM, Gajula SS (2022). The Commonwealth Caribbean COVID-19: regions resilient pathway during pandemic. Front Public Health.

[9] Sallam M (2021). COVID-19 vaccine hesitancy worldwide: a concise systematic review of vaccine acceptance rates. Vaccines.

[10] Umakanthan S, Lawrence S (2022). Predictors of COVID-19 vaccine hesitancy in Germany: a cross-sectional, population-based study. Postgrad Med J.

[11] Umakanthan S, Patil S, Subramaniam N, Sharma R (2021). COVID-19 vaccine hesitancy and resistance in India explored through a population-based longitudinal survey. Vaccines Basel.

[12] Page MJ, McKenzie JE, Bossuyt PM, Boutron I, Hoffmann TC, Mulrow CD (2021). The PRISMA 2020 statement: an updated guideline for reporting systematic reviews. BMJ.

[13] Hopewell S, McDonald S, Clarke M, Egger M (2007). Grey literature in meta-analyses of randomized trials of health care interventions. Cochrane Database Syst Rev.

[14] Zarifkar P, Kamath A, Robinson C, Morgulchik N, Shah SFH, Cheng TKM (2021). Clinical characteristics and outcomes in patients with COVID-19 and cancer: a systematic review and meta-analysis. Clin Oncol R Coll Radiol G B.

[15] Ouzzani M, Hammady H, Fedorowicz Z, Elmagarmid A (2016). Rayyan—a web and mobile app for systematic reviews. Syst Rev.

[16] Study Quality Assessment Tools | NHLBI, NIH [Internet]. [cited 2022 May 30]. https://www.nhlbi.nih.gov/health-topics/study-quality-assessment-tools

[17] Lin L, Xu C (2020). Arcsine-based transformations for meta-analysis of proportions: pros, cons, and alternatives. Health Sci Rep.

[18] Egger M, Davey Smith G, Schneider M, Minder C (1997). Bias in meta-analysis detected by a simple, graphical test. BMJ.

[19] Roland M, Abdelhafidh LB, Déom V, Vanbiervliet F, Coppieters Y, Racapé J (2021). SARS-CoV-2 screening among people living in homeless shelters in Brussels, Belgium. PLoS ONE.

[20] Cha S, Henry A, Montgomery MP, Laws RL, Pham H, Wortham J (2021). Morbidity and mortality among adults experiencing homelessness hospitalized with COVID-19. J Infect Dis.

[21] Gaeta JM, De Las ND, Munson DG, Barocas JA, Walsh KE (2020). Case 21–2020: a 66-year-old homeless man with covid-19. N Engl J Med.

[22] Tobolowsky FA (2020). COVID-19 outbreak among three affiliated homeless service sites—King County, Washington, 2020. MMWR Morb Mortal Wkly Rep.

[23] Ghalamkarpour F, Pourani MR, Abdollahimajd F, Zargari O (2022). A case of severe psoriatic erythroderma with COVID-19. J Dermatol Treat.

[24] Fields VL, Kiphibane T, Eason JT, Hafoka SF, Lopez AS, Schwartz A (2021). Assessment of contact tracing for COVID-19 among people experiencing homelessness, Salt Lake County Health Department, March–May 2020. Ann Epidemiol.

[25] Imbert E, Kinley PM, Scarborough A, Cawley C, Sankaran M, Cox SN (2020). Coronavirus disease 2019 (COVID-19) outbreak in a San Francisco homeless shelter. Clin Infect Dis.

[26] Przydzial P, Tchomobe G, Amin K, Engell E, Okoh AK (2020). COVID-19 crossing paths with AIDS in the homeless. J Med Virol.

[27] Byrnes S, Bisen M, Syed B, Huda S, Siddique Z, Sampat P (2020). COVID-19 encephalopathy masquerading as substance withdrawal. J Med Virol.

[28] Kiran T, Craig-Neil A, Das P, Lockwood J, Wang R, Nathanielsz N (2020). Mobile outreach testing for COVID-19 in twenty homeless shelters in Toronto, Canada. medRxiv.

[29] Redditt V, Wright V, Rashid M, Male R, Bogoch I (2020). Outbreak of SARS-CoV-2 infection at a large refugee shelter in Toronto, April 2020: a clinical and epidemiologic descriptive analysis. CMAJ Open.

[30] Baggett TP, Keyes H, Sporn N, Gaeta JM (2020). Prevalence of SARS-CoV-2 infection in residents of a large homeless shelter in Boston. JAMA.

[31] Ghinai I, Davis ES, Mayer S, Toews KA, Huggett TD, Snow-Hill N (2020). Risk factors for severe acute respiratory syndrome coronavirus 2 infection in homeless shelters in Chicago, Illinois—March–May, 2020. Open Forum Infect Dis.

[32] McCormick D, Scott T, Chavez J, Wilcox K, Marx GE, Stella SA (2020). LB-12 SARS-CoV-2 RNA and antibodies among people experiencing homelessness and staying in shelters or outdoor encampments in Denver, Colorado, May-July 2020. Open Forum Infect Dis.

[33] Prodanuk M, Wagner S, Orkin J, Noone D (2021). Social vulnerability and COVID-19: a call to action for paediatric clinicians. Paediatr Child Health.

[34] Samuels EA, Karb R, Vanjani R, Trimbur MC, Napoli A (2020). Congregate Shelter Characteristics and Prevalence of Asymptomatic SARS-CoV-2. medRxiv.

[35] Kuhn R, Henwood B, Lawton A, Kleva M, Murali K, King C (2021). COVID-19 vaccine access and attitudes among people experiencing homelessness from pilot mobile phone survey in Los Angeles, CA. PLoS ONE.

[36] Iacoella C, Ralli M, Maggiolini A, Arcangeli A, Ercoli L (2021). Acceptance of COVID-19 vaccine among persons experiencing homelessness in the City of Rome. Italy Eur Rev Med Pharmacol Sci.

[37] Knight KR, Duke MR, Carey CA, Pruss G, Garcia CM, Lightfoot M (2021). “This is about the coolest thing I’ve ever seen is that you just showed right up.” COVID-19 testing and vaccine acceptability among homeless-experienced adults: Qualitative data from two samples. medRxiv.

[38] Ekezie W, Czyznikowska BM, Rohit S, Harrison J, Miah N, Campbell-Morris P (2020). The views of ethnic minority and vulnerable communities towards participation in COVID-19 vaccine trials. J Public Health Oxf Engl..

[39] Claude KM, Serge MS, Alexis KK, Hawkes MT (2020). Prevention of COVID-19 in internally displaced persons camps in War-Torn North Kivu, Democratic Republic of the Congo: a mixed-methods study. Glob Health Sci Pract.

[40] Deal A, Hayward SE, Huda M, Knights F, Crawshaw AF, Carter J (2021). Strategies and action points to ensure equitable uptake of COVID-19 vaccinations: a national qualitative interview study to explore the views of undocumented migrants, asylum seekers, and refugees. J Migr Health.

[41] Longchamps C, Ducarroz S, Crouzet L, Vignier N, Pourtau L, Allaire C (2021). COVID-19 vaccine hesitancy among persons living in homeless shelters in France. Vaccine.

[42] Baggett TP, Keyes H, Sporn N, Gaeta JM (2020). COVID-19 outbreak at a large homeless shelter in Boston: Implications for universal testing. medRxiv.

[43] Baggett TP, Scott JA, Le MH, Shebl FM, Panella C, Losina E, et al. Clinical outcomes, costs, and cost-effectiveness of strategies for adults experiencing sheltered homelessness during the COVID-19 pandemic. JAMA Netw Open. 3(12):e2028195; 2020 10.1001/jamanetworkopen.2020.28195 PMC7756240 33351082

[44] Le Bihan C, Faucherre V, Le Moing V, Mehenni A, Nantes D, Da Silva A (2021). COVID-19: The forgotten cases of hidden exiles. Infect Dis Now.

[45] Lindner AK, Sarma N, Rust LM, Hellmund T, Krasovski-Nikiforovs S, Wintel M (2021). Monitoring for COVID-19 by universal testing in a homeless shelter in Germany: a prospective feasibility cohort study. BMC Infect Dis.

[46] Maki G, Bowser D, Shallal A, Prentiss T, Zervos M, Rehman NK (2020). Detroit’s response to COVID-19 in homeless shelters. Open Forum Infect Dis.

[47] Marquez H, Ramers C, Northrup A, Tam A, Liu J, Rojas S (2020). Response to the COVID-19 pandemic among people experiencing homelessness in congregant living settings in San Diego, CA. Clin Infect Dis.

[48] Alarcón J, Khan TV (2021). Adapting backpack medicine in COVID-19 response for people experiencing homelessness in Southern California. Am J Public Health.

[49] Kelly D, Murphy H, Vadlamudi R, Kraut R, Dalessio K, Malani AN (2021). Successful public health measures preventing coronavirus disease 2019 (COVID-19) at a Michigan homeless shelter. Infect Control Hosp Epidemiol.

[50] Baggett TP, Racine MW, Lewis E, De Las ND, O’Connell JJ, Bock B (2020). Addressing COVID-19 among people experiencing homelessness: description, adaptation, and early findings of a multiagency response in Boston. Public Health Rep.

[51] Barocas JA, Gai MJ, White LF, Faretra D, Sachs K, Komaromy M (2021). Implementation of a recuperation unit and hospitalization rates among people experiencing homelessness with COVID-19. JAMA Netw Open.

[52] Komaromy M, Harris M, Koenig RM, Tomanovich M, Ruiz-Mercado G, Barocas JA (2020). Caring for COVID’s most vulnerable victims: a safety-net hospital responds. Res Sq..

[53] Komaromy M, Tomanovich M, Taylor JL, Ruiz-Mercado G, Kimmel SD, Bagley SM (2021). Adaptation of a system of treatment for substance use disorders during the COVID-19 pandemic. J Addict Med.

[54] Dotson S, Ciarocco S, Koh KA (2020). Disaster psychiatry and homelessness: creating a mental health COVID-19 response. Lancet Psychiatry.

[55] Ramírez-Cervantes KL, Romero-Pardo V, Pérez-Tovar C, Martínez-Alés G, Quintana-Diaz M (2020). A medicalized hotel as a public health resource for the containment of Covid-19: more than a place for quarantining. J Public Health Oxf Engl..

[56] Bharmal A. Find and Treat: taking health care on to the streets of London | The King’s Fund. https://www.kingsfund.org.uk/blog/2021/02/find-and-treat-taking-health-care-streets-london

[57] Harris M, Johnson S, Mackin S, Saitz R, Walley AY, Taylor JL (2020). Low barrier tele-buprenorphine in the time of COVID-19: a case report. J Addict Med.

[58] Parkes T, Carver H, Masterton W, Falzon D, Dumbrell J, Grant S (2021). ‘They already operated like it was a crisis, because it always has been a crisis’: a qualitative exploration of the response of one homeless service in Scotland to the COVID-19 pandemic. Harm Reduct J.

[59] Roncero C, García-Ullán L, de la Iglesia-Larrad JI, Martín C, Andrés P, Ojeda A (2020). The response of the mental health network of the Salamanca area to the COVID-19 pandemic: the role of the telemedicine. Psychiatry Res.

[60] Aguilar L, Vicente-Hernández B, Remón-Gallo D, García-Ullán L, Valriberas-Herrero I, Maciá-Casas A (2021). A real-world ten-week follow-up of the COVID outbreak in an outpatient drug clinic in Salamanca (Spain). J Subst Abuse Treat.

[61] Roncero C, Vicente-Hernández B, Casado-Espada NM, Aguilar L, Gamonal-Limcaoco S, Garzón MA (2020). The impact of COVID-19 pandemic on the castile and leon addiction treatment network: a real-word experience. Front Psychiatry.

[62] Caswell R, Battye F. Homelessness and the response to COVID-19: learning from lockdown: Data and data sharing: Final report [Internet]. The Strategy Unit; NHS Midlands and Lancashire Commisioning Support Unit; 2021. https://www.strategyunitwm.nhs.uk/sites/default/files/2021-02/Homelessness%20data%20and%20data%20sharing%20Strategy%20Unit%20Final%20Report.pdf

[63] Callaghan D, Cope G, Battye F. Homelessness and the response to COVID-19: learning from lockdown: Final report [Internet]. The Strategy Unit; NHS Midlands and Lancashire Commisioning Support Unit; 2021. https://www.strategyunitwm.nhs.uk/sites/default/files/2021-02/Homelessness%20and%20the%20response%20to%20COVID-19%20Strategy%20Unit%20Final%20Report%20%20%20%281%29.pdf

[64] Ferguson JM, Jacobs J, Yefimova M, Greene L, Heyworth L, Zulman DM (2020). Virtual care expansion in the Veterans Health Administration during the COVID-19 pandemic: clinical services and patient characteristics associated with utilization. J Am Med Inform Assoc JAMIA.

[65] Heflin KJ, Gillett L, Alexander A (2020). Lessons from a free clinic during covid-19: medical students serving individuals experiencing homelessness using tele-health. J Ambulatory Care Manage.

[66] Benjenk I, Saliba Z, Duggal N, Albaroudi A, Posada J, Chen J (2021). Impact of COVID-19 mitigation efforts on adults with serious mental illness. J Nerv Ment Dis.

[67] Verity A, Naidu D, Tzortziou-Brown V (2020). Does total triage and remote-by-default consulting impact vulnerable groups: a pilot study. medRxiv.

[68] Coombs J, Gray T. Lessons learnt from councils’ response to rough sleeping during the COVID-19 pandemic | Local Government Association [Internet]. Local Government Association; 2020. https://www.local.gov.uk/publications/lessons-learnt-councils-response-rough-sleeping-during-covid-19-pandemic

[69] Comptroller and Auditor General. Investigation into the housing of rough sleepers during the COVID-19 pandemic [Internet]. Ministry of Housing, Communities & Local Government; 2021. https://www.nao.org.uk/wp-content/uploads/2021/01/Investigation-into-the-housing-of-rough-sleepers-during-the-COVID-19-pandemic.pdf

[70] Cromarty H. Coronavirus: support for rough sleepers (England). House of Commons Library; 2021 Oct. (Number 9057).

[71] Cookson E, Orchard B. Housing and health: working together to respond to rough sleeping during Covid-19 [Internet]. St Mungo’s; 2021. https://www.mungos.org/app/uploads/2021/01/St-Mungos-Housing-and-Health-Report.pdf

[72] Fitzpatrick S, Watts B, Pawson H, Bramley G, Wood J, Stephens M. The homelessness monitor: England 2021 [Internet]. Crisis; 2021 Mar p. 74. https://www.crisis.org.uk/media/244702/crisis-england-monitor-2021.pdf

[73] Marcus TS, Heese J, Scheibe A, Shelly S, Lalla SX, Hugo JF (2020). Harm reduction in an emergency response to homelessness during South Africa’s COVID-19 lockdown. Harm Reduct J.

[74] Fitzpatrick S, Watts B, Sims R. Homelessness Monitor England 2020: COVID-19 Crisis Response Briefing [Internet]. Crisis; 2020 p. 26. https://www.crisis.org.uk/media/242907/homelessness_monitor_england_2020_covid19_crisis_response_briefing.pdf

[75] Nazroo PJ, Murray K, Taylor H, Bécares DL, Field Y, Kapadia DD, et al. Rapid evidence review: inequalities in relation to COVID-19 and their effects on London. 2020;80.

[76] An unsafe distance: the impact of the COVID-19 pandemic on excluded people in England [Internet]. Doctors of the World. 2020. https://www.doctorsoftheworld.org.uk/news/covid19-rapid-needs-assessment/

[77] Whitehead C, Edge A, Rotolo M. Homelessness and rough sleeping in the time of COVID-19. 46.

[78] Pennington J, Rich H. Homeless and Forgotten: Surviving lockdown in temporary accommodation [Internet]. Shelter; 2020 Dec p. 31. https://assets.ctfassets.net/6sxvmndnpn0s/117BUNn9h9puF7ATy9TVV3/134b941d0fa8d161c9890e285f431417/TA_report_FINAL_PDF.pdf

[79] Left Out: Supporting homeless young people during and beyond the COVID-19 pandemic [Internet]. Centrepoint; 2020. https://centrepoint.org.uk/media/4623/left-out-centrepoint-covid-and-rough-sleeping-report.pdf

[80] A year like no other: youth homelessness during the COVID pandemic [Internet]. Centrepoint; https://centrepoint.org.uk/media/4773/a-year-like-no-other.pdf

[81] The Impact of Covid-19 on Homeless Service Providers & Homeless People: The Migrant Perspective [Internet]. European Federation of National Organisations Working with the Homeless; 2021 Feb [cited 2022 May 31]. https://www.feantsa.org/en/report/2021/03/05/the-impact-of-covid-19-on-homeless-service-providers-homeless-people-the-migrant-perspective?bcParent=27

[82] Koh D (2020). Migrant workers and COVID-19. Occup Environ Med.

[83] Homelessness, Access to services and COVID- 19: Learning during the pandemic to inform our future [Internet]. Healthcare Improvement Scotland; 2020 Dec. https://ihub.scot/media/7709/20210127-homelessness-access-to-services-and-covid-19-learning-during-the-pandemic-to-inform-our-future.pdf

[84] Benavides AD, Nukpezah JA (2020). How local governments are caring for the homeless during the COVID-19 pandemic. Am Rev Public Adm.

[85] Irwin M, Amanuel Y, Bickers B, Nguyen MA, Russell OW (2021). Impacts of the COVID-19 pandemic on preexisting racial and ethnic disparities, and results of an integrated safety net response in Arlington County, Virginia. Health Secur..

[86] Gurvinder PS, Arun P, Chavan BS (2020). Migrant workers’ needs and perceptions while lodged in a shelter home in India during the COVID-19 pandemic. Prim Care Companion CNS Disord..

[87] McIntyre K, Castillo EM, Aminlari A, Kreshak AA. Use of a hotel for an emergency department homeless population requiring quarantine for SARS-CoV-2. Acad Emerg Med. 2021. 10.1111/acem.14249?af=R

[88] Fuchs JD, Carter HC, Evans J, Graham-Squire D, Imbert E, Bloome J (2021). Assessment of a hotel-based COVID-19 isolation and quarantine strategy for persons experiencing homelessness. JAMA Netw Open.

[89] Ingle TA, Morrison M, Wang X, Mercer T, Karman V, Fox S (2021). Projecting COVID-19 isolation bed requirements for people experiencing homelessness. PLoS ONE.

[90] Chapman LAC, Kushel M, Cox SN, Scarborough A, Cawley C, Nguyen TQ (2021). Comparison of infection control strategies to reduce COVID-19 outbreaks in homeless shelters in the United States: a simulation study. BMC Med.

[91] Nande A, Sheen J, Walters EL, Klein B, Chinazzi M, Gheorghe AH (2021). The effect of eviction moratoria on the transmission of SARS-CoV-2. Nat Commun.

[92] Foster-Bey LD, Eid AE, Darian MA (2021). Washing without a sink. Acad Med.

[93] Montgomery MP, Carry MG, Garcia-Williams AG, Marshall B, Besrat B, Bejarano F (2021). Hand hygiene during the COVID-19 pandemic among people experiencing homelessness—Atlanta, Georgia, 2020. J Commun Psychol.

[94] Davies SH, Della Porta A, Renjilian CB, Sit L, Ginsburg KR (2020). Lessons learned: achieving critical mass in masking among youth in congregate living. J Adolesc Health.

[95] Steer KJD, Klassen DC, O’Gorman CM, Webster M, Mitchell M, Krichevsky L (2021). Cups for COVID: rapid implementation of a harm reduction initiative to support populations experiencing homelessness during the COVID-19 pandemic. Can J Public Health Rev Can Santé Publique.

[96] O’Carroll A, Duffin T, Collins J (2021). Harm reduction in the time of COVID-19: Case study of homelessness and drug use in Dublin, Ireland. Int J Drug Policy.

[97] Alavi M, Moghanibashi-Mansourieh A, Radfar SR, Alizadeh S, Bahramabadian F, Esmizade S (2021). Coordination, cooperation, and creativity within harm reduction networks in Iran: COVID-19 prevention and control among people who use drugs. Int J Drug Policy.

[98] Honorato BEF, Oliveira ACS (2020). Homeless population and COVID-19. Rev Adm Pública.

[99] Jain V, Schwarz L, Lorgelly P (2021). A rapid review of COVID-19 vaccine prioritization in the US: alignment between Federal guidance and State practice. medxRiv..

[100] Yu X, Li M, Lawson-Portuphy L, Chowdhury A, Badrinath P (2021). Comparative analysis of variation in the quality and completeness of local outbreak control plans for SARS-CoV-2 in English local authorities. J Public Health.

[101] Sheleme T, Bekele F, Ayela T (2020). Clinical presentation of patients infected with coronavirus disease 19: a systematic review. Infect Dis.

[102] Fazel S, Geddes JR, Kushel M (2014). The health of homeless people in high-income countries: descriptive epidemiology, health consequences, and clinical and policy recommendations. Lancet Lond Engl.

[103] Miyawaki A, Hasegawa K, Tsugawa Y (2020). Lessons from influenza outbreaks for potential impact of COVID-19 outbreak on hospitalizations, ventilator use, and mortality among homeless persons in New York State. J Gen Intern Med.

[104] Dhama K, Sharun K, Tiwari R, Dhawan M, Emran TB, Rabaan AA (2021). COVID-19 vaccine hesitancy-reasons and solutions to achieve a successful global vaccination campaign to tackle the ongoing pandemic. Hum Vaccines Immunother.

[105] Detoc M, Bruel S, Frappe P, Tardy B, Botelho-Nevers E, Gagneux-Brunon A (2020). Intention to participate in a COVID-19 vaccine clinical trial and to get vaccinated against COVID-19 in France during the pandemic. Vaccine.

[106] Reiter PL, Pennell ML, Katz ML (2020). Acceptability of a COVID-19 vaccine among adults in the United States: how many people would get vaccinated?. Vaccine.

[107] Inc G. U.S. Readiness to Get COVID-19 Vaccine Steadies at 65% [Internet]. Gallup.com. 2021 https://news.gallup.com/poll/328415/readiness-covid-vaccine-steadies.aspx

[108] Hayawi K, Shahriar S, Serhani MA, Taleb I, Mathew SS (2022). ANTi-Vax: a novel Twitter dataset for COVID-19 vaccine misinformation detection. Public Health.

[109] Farrell SJ, Dunn M, Huff J (2020). Examining health literacy levels in homeless persons and vulnerably housed persons with mental health disorders. Commun Ment Health J..

[110] Dzigbede KD, Gehl SB, Willoughby K (2020). Disaster resiliency of US local governments: insights to strengthen local response and recovery from the COVID-19 pandemic. Public Adm Rev.

