# Supplementary material for: COVID-19 in the homeless population: a scoping review and meta-analysis examining differences in prevalence, presentation, vaccine hesitancy and government response in the first year of the pandemic
Source: BMC Infect Dis. 2023 Mar 14;23:155. doi: 10.1186/s12879-023-08037-x (PMC10012317; doi:10.1186/s12879-023-08037-x)
Supplement: Supplementary file 1 — Additional file 1. Table of search terms. [file 12879_2023_8037_MOESM1_ESM.docx]

**Appendix A**

The specific search strategy implemented across databases is listed below.

[ *“2019 novel coronavirus” OR “COVID-19” OR “COVID” OR “2019-nCov” OR “corona” OR “covid*” OR “sars” OR “SARS” OR “severe acute respiratory syndrome” OR "severe acute*

*respiratory syndrome coronavirus 2" OR “coronavirus 2019” OR “SARS-CoV-2”* ]

*AND*

[*“homelessness” OR “homeless*” OR “homeless” OR “roofless” OR “homeless persons” OR “street” OR “street dwellers” OR “no fixed abode/home” OR “houseless” OR “rough sleepers”*]

In databases where exploding term (*) was not possible, the following search terms were used:

[*“2019 novel coronavirus” OR “COVID-19” OR “COVID” OR “2019-nCov” OR “corona” OR “sars” OR “SARS” OR “severe acute respiratory syndrome” OR "severe acute*

*respiratory syndrome coronavirus 2" OR “coronavirus 2019” OR “SARS-CoV-2”* ]

*AND*

[*“homelessness” OR “homeless” OR “roofless” OR “homeless persons” OR “street” OR “street dwellers” OR “no fixed abode/home” OR “houseless” OR “rough sleepers”*]
