# Supplementary material for: COVID-19 in the homeless population: a scoping review and meta-analysis examining differences in prevalence, presentation, vaccine hesitancy and government response in the first year of the pandemic
Source: BMC Infect Dis. 2023 Mar 14;23:155. doi: 10.1186/s12879-023-08037-x (PMC10012317; doi:10.1186/s12879-023-08037-x)
Supplement: Supplementary file 2 — Additional file 2. List of databases searched. [file 12879_2023_8037_MOESM2_ESM.docx]

**Appendix B**

The following databases, including grey literature databases, were searched:

- PubMed
- Embase / OVID
- Covid-19 registry Cochrane
- Global Health (OVID)
- HMIC (OVID)
- PsychEXTRA (APA) (OVID)
- OpenDOAR
- Social Care Online
- Social Science Research Network
- MedrXiv
- BiorXiv
- WHO website
- Public Health England
- FEANTSA
- EUPHA
- Clinicaltrials.gov
- WHO Coronavirus Disease
- Web of Science
- EU Register Trials
- NIHR Be Part of Research

The literature terms used to search are specified in Appendix A - these were individually inputted into grey literature databases at the discretion of the researcher.
