# Supplementary figures and images for: COVID-19 in the homeless population: a scoping review and meta-analysis examining differences in prevalence, presentation, vaccine hesitancy and government response in the first year of the pandemic
Source: BMC Infect Dis. 2023 Mar 14;23:155. doi: 10.1186/s12879-023-08037-x (PMC10012317; doi:10.1186/s12879-023-08037-x)

**Appendix C**

Funnel plots for the studies included in meta-analysis are presented below

**
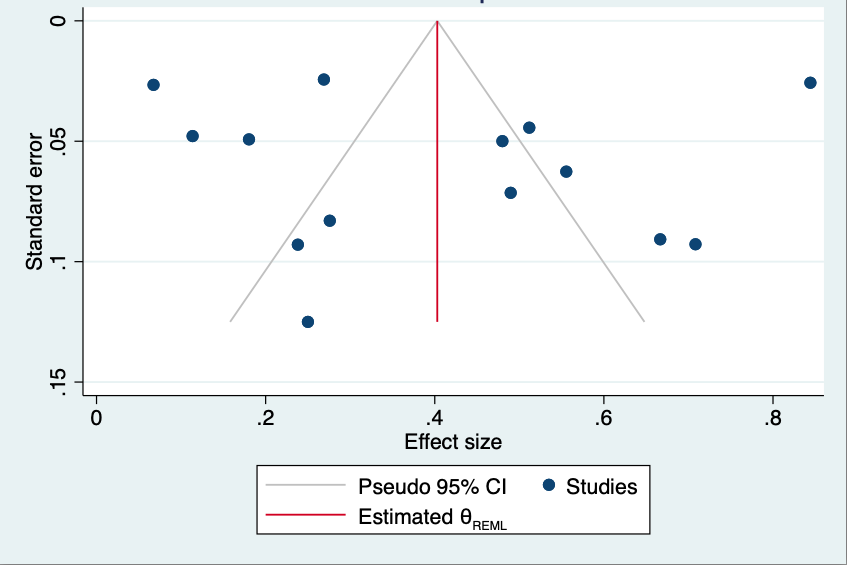
**

Supplement: Supplementary file 3 — Additional file 3. Funnel plots for meta-analysis. [file 12879_2023_8037_MOESM3_ESM.docx]
