# Supplementary material for: COVID-19 in the homeless population: a scoping review and meta-analysis examining differences in prevalence, presentation, vaccine hesitancy and government response in the first year of the pandemic
Source: BMC Infect Dis. 2023 Mar 14;23:155. doi: 10.1186/s12879-023-08037-x (PMC10012317; doi:10.1186/s12879-023-08037-x)
Supplement: Supplementary file 4 — Additional file 4. Characteristics of studies describing vaccine hesitancy. [file 12879_2023_8037_MOESM4_ESM.docx]

**Appendix D: Study characteristics of vaccine hesitancy**

|  | **Author** | **City (Country), study period** | **Total sample size** | **Population**  **(FEANTSA definition)** | **When/how vaccine hesitancy were assessed** | **Mean age** | **Male sex, n (%)** |
| --- | --- | --- | --- | --- | --- | --- | --- |
| JR43 | Deal et al. 2021 | UK | 32 | Migrant, asylum seekers and refugees in temporary accommodation (houseless) | Semi structured qualitative interview to assess COVID19 vaccine hesitancy | 37.1 (mean age) | 11 (34%) |
| JR21 | Claude, K et al 2020 | Democratic Republic of Congo | 187 | Internally displaced persons camps  (inadequate) | Survey questionnaires (n=164) and qualitative focus group discussions (n=23) | 43 (IQR = 28-58) for the survey questionnaire participants | 90 (54.9%) |
| ME17 | Ekenzie, W et al 2021 | UK (East Midlands) | 15 | ‘Homeless’ amongst other groups including Gypsy, Roma and Travellers communities  (unclear) | Qualitative investigation via semi-structured interviews | N/A | N/A |
| EA48 | Longchamps, C et al 2021 | France (Paris and Lyon) | 235 | Persons living in homeless shelters  (houseless) | Questionnaire | N/A | 66.30% |
| EA3 | Kuhn,R et al 2021 | Los Angeles, USA | 90 | Persons experiencing homelessness (houseless, roofless, insecure) | Mobile phone surveys | 48.7 (mean age) | 41% |
| TA27 | Knight KR et al. 2021 | Oakland and San Francisco, USA | 94 | Recruited from HOPE HOME study and unsheltered population from testing outreach program  (houseless, roofless, insecure) | Qualitative telephone interviews | 59 in Testing outreach, 62 in HOPE HOME | 39 (72%) and 20 (54) respectively |
| HG27 | Iacoella, C et al 2021 | Vatican City state, Italy | 112 | Homeless people referring to a local primary care medical facility for a routine check up  (unclear) | Questionnaire | 53.12 (range 22-79 years) | 85 (75.9%) |

Title: Characteristics of studies looking at vaccine hesitancy
