# Supplementary material for: COVID-19 in the homeless population: a scoping review and meta-analysis examining differences in prevalence, presentation, vaccine hesitancy and government response in the first year of the pandemic
Source: BMC Infect Dis. 2023 Mar 14;23:155. doi: 10.1186/s12879-023-08037-x (PMC10012317; doi:10.1186/s12879-023-08037-x)
Supplement: Supplementary file 5 — Additional file 5. Characteristics of studies describing policies (non-pharmaceutical interventions). [file 12879_2023_8037_MOESM5_ESM.docx]

**Appendix E: Study characteristics of policies (non-pharmaceutical interventions)**

*Mass Testing*

| **Paper ID** | **Author** | **City (Country), study period** | **Population**  **(FEANTSA definition)** | **Policy implemented** | **How Policy Was Assessed** | **Age** | **Sex** | **Limitations** | **Main Conclusion** |
| --- | --- | --- | --- | --- | --- | --- | --- | --- | --- |
| TA2 | Gaeta et al., 2020 (21) | Boston (USA), 2020 | Shelter only (houseless) | Universal SARS-CoV-2 testing upon identification of a COVID-19 case cluster, and universal testing subsequently every two weeks at congregate shelters. Creating alternative care sites (isolation and quarantine) | Identification of number of Covid-19 cases | N/A | N/A | N/A | Symptom screening alone will miss a large number of cases when community transmission is mounting or a shelter-based cluster of Covid-19 cases has been identified. Collaborative efforts must ensure that adequate isolation and quarantine sites are developed |
| TA9 | Baggett et al., 2020 (42) | Boston (USA), March 2020 | Shelter only (houseless) | Universal SARS-CoV-2 PCR testing and symptom assessment of an adult homeless shelter population in Boston shortly after identification of a COVID-19 case cluster | Number of individuals who were PCR-positive for SARS-CoV-2 and/or symptomatic | Mean 51.6 years | 71.6% Male | N/A | Front-door symptom screening in homeless shelter settings will likely miss a substantial number of COVID-19 cases in this population. Universal PCR testing approach for identifying COVID-19 among people experiencing homelessnessness is recommended. |
| TA47 | Baggett et al., 2020 (50) | Boston (USA), July-August 2020 | Shelter only  (houseless) | Implementation of COVID-19 care model: symptom screening at shelters, expedited testing at pop-up sites, isolation and management venues, quarantine venues, contact investigation and tracing. Identified emerging COVID-19 cluster and adapted in response to COVID-19 outbreak | Reported by mass testing of homeless people | N/A | N/A | Further modifications needed including universal testing and innovative housing strategies among this vulnerable population. | In response to COVID-19 outbreak, symptom screening was de-emphasised given the high number of asymptomatic or minimally symptomatic infections discovered during mass testing, contact tracing and quarantining phased out, isolation and management venues rapidly expanded. Universal testing is a focal point of ongoing efforts to mitigate the effect of COVID-19 in the homeless population. |
| AR9 | Baggett et al., 2020 (43) | Boston, USA, 2020 | Sheltered homeless (houseless) | Microsimulation model of eight different scenarios, including daily symptom screening within isolation areas, and provision of alternative care sites (ACSs) | Data from national database and literature along with development of microsimulation model | N/A | N/A | Exclusion of adults experiencing homelessness as part of a family, and unsheltered homelessness individuals. Assumption of homogenous mixing of adults experiencing sheltered homelessness. No assumption of increased comorbidities amongst the homeless population. | Compared to no intervention, daily symptom screening with isolation areas for those with pending tests or confirmed COVID-19 and moderate disease leads to 37% fewer infections and 46% lower costs. Addition of universal PCR testing every 2 weeks in a surging pandemic decreases infections at a reasonable cost. Thus daily symptom screening with PCR testing of individuals who had positive screening results and ACS-based COVID-19 management was the most efficient strategy and was cost-saving relative to no intervention. Strategies using ACSs for isolation of symptomatic individuals were associated with substantially decreased costs. |
| EA52 | Le Bihan et al., 2020 (44) | Montpellier (France), January 2020 | ‘Squats’ (insecure) | Two different mass testing strategies in two different ‘squats’ in response to diagnosis of symptomatic COVID cases: one-off PCR test in one, and screening sessions over a period of 14 days in the other squat. | Policy was assessed by reports and numbers of individuals who came forward to be tested, individuals in response by reports of asymptomatic spread that was highly endemic in one of the squats but not the other. | N/A | N/A | N/A | In squat A: 50 (25%) persons screened, 19 were found positive, 3 accepted a transfer. In squat B: 65 (54%) persons screened at three different times, 2 found positive.  Mass testing irrespective of disease symptoms is an important strategy regardless of symptoms status; however acceptance amongst the population was low. Thus discrepant outcomes may reflect different levels of sanitation, prevention and acceptance of interventions. |
| ME1 | Lindner et al., 2020 (45) | Berlin, (Germany), October 2020 | Shelter only (houseless) | Extracted from self-collected naso-oropharyngeal swabs in bed homeless shelter. | Policy was assessed by reports of large self-collected testing schemes where none came out positive. | N/A | N/A | Repeat attempts were required in order to collect the samples and significant assistance was needed to collect the samples. | Large mass testing scheme is possible and can be successful in homeless shelters, however it notes that the major barriers are laboratory capacity and human resources. |
| ME2 | Kiran et al., 2020 (28) | Toronto (Canada), November 2020 | Shelter only  (houseless) | Extracted from self-collected naso-oropharyngeal swabs done at different locations across the city. | Evaluation of factors linked with positivity in the mass testing results. | N/A | N/A | N/A | Asymptomatic testing in homeless shelters in the context of an outbreak is an important strategy to adopt in shelters. However, this strategy was less supportive of surveillance testing without a known case present in the shelter. |
| SM2 | Maki et al., 2020 (46) | Detroit, USA, 2020 | Shelter Only (houseless) | Screening of homeless residents in shelters | Monitoring of temperature and symptoms of residents across 24 shelters in Detroit and universal COVID-19 testing was performed across 13 other shelters | N/A | N/A | N/A | Increased screening and advanced prevention based policies are important in shelters since they are at higher risk of outbreaks |
| ME5 | Marquez et al., 2020 (47) | San Diego (USA), April-August 2020 | Sheltered and unsheltered individuals (houseless, roofless) | Within temporary accommodation sites, pre-emptive testing strategy combined with isolation and symptom screening | Prevalence of COVID-19 within the tested population | N/A | N/A | N/A | Preemptive testing strategy in congregant living settings, combined with accessible isolation of individuals found to be positive and consistent symptom screening of individuals found to be negative may be sufficient to avoid large outbreaks among PEH |
| TA44 | Alarcon et al., 2021 (48) | Ventura County, California (USA), December 2021 | Homeless encampments, homeless shelters, rough sleepers Housing (houseless, roofless, insecure) | Temporary housing and mass testing across three different cities in California. | Mass testing of 150 people and assistance of temporary housing for 400 people assisting in the prevention of outbreaks. | N/A | N/A | N/A | Mass testing and temporary housing to accommodate homeless individuals in times of COVID-19 pandemic aids in preventing outbreaks in this vulnerable population and in provision of healthcare. |
| JR46 | Kelly et al., 2020 (49) | Michigan (USA), March-April 2020 | Homeless shelter (houseless) | Triaging PEH based on COVID status before entering shelter. Use of isolation ‘emergency’ rooms for those with COVID. Use of sheltering in place, temporary pay and increase of staff respecting mandated masks and covid testing. | Assessed via universal screenings conducted where only 2 positive cases of COVID were present | N/A | N/A | N/A | Emergency housing with measures put in place is an effective way to shelter homeless individuals and lower the risk of COVID infection |

*Adaptation of Healthcare Service Provision*

| **Paper ID** | **Author** | **City (Country), study period** | **Population**  **(FEANTSA definition)** | **Policy implemented** | **How Policy Was Assessed** | **Age** | **Sex** | **Limitations** | **Main Conclusion** |
| --- | --- | --- | --- | --- | --- | --- | --- | --- | --- |
| KS35 | Barocas et al, 2021 (51) | Boston (USA), March 2021 | Identification of homeless inpatients (houseless) | COVID-19 Recuperation Unit helped homeless people isolate and get treatment for substance abuse leading to a 28% reduction in COVID hospitalisations | Reported by patients in this COVID-19 Recuperation Unit in Boston. | N/A | N/A | Limitation is that the hospitalisations that were looked at was only one hospital, therefore potentially missing some cases. | The COVID-19 Recuperation Unit was associated with a decrease in hospitalisation among PEH with COVID-19, by providing isolation and quarantine space. |
| TA1 | Komaromy et al, 2020 (52) | Boston, (USA), March-April 2020 | Identification of homeless inpatients (houseless) | COVID-19 Recuperation Unit to help homeless people isolate and get treatment for any type of mental and substance use disorder. | Reported by patients in this COVID-19 Recuperation Unit in New England. | N/A | N/A | N/A | CRU helped alleviate pressure of bed space and avoid exceeding hospital bed capacity during the epidemic surge. Provided mental health, substance use disorder and addictions support thus emphasising CRU can provide safe and effective medical respite for PEH. |
| TA43 | Komaromy et al., 2020 (53) | Boston (USA), December 2020 | Temporary accommodation i.e. recovery unit shelters (houseless) | Measures at a centre for people with substance use disorders (SUDs): switch to telehealth, simultaneous launch of COVID-19 Recuperation Unit for homeless population, text messaging, mobile-health outreach clinic on those with substance use disorders | Commentary of program and effectiveness | N/A | N/A | N/A | COVID-19 recovery unit created an environment that provided a harm-reduction approach to SUDs and immediate medical assistance. Good rapid initiation of medication treatment. Good response to telehealth initiatives to provide services. |
| EA18 | Dotson et al., 2020 (54) | Boston, (USA), April-May 2020 | “homeless patients” with mention of previously sheltered  (Unclear) | Implementation of mental health disaster response unit where 500 beds were created to respond to mental health challenges in the homeless population through counselling services and other measures. | Reports of anecdotal evidence from patients that said they benefited from this service. | N/A | N/A | N/A | Mental health disaster response unit in Boston with provision of services to provide to homeless individuals with mental health issues is beneficial. |
| HG17 | Ramirez-Cervantes et al., 2020 (55) | Madrid (Spain)., March-May 2020 | Unsheltered, amongst wider population including migrants (roofless, inadequate) | Implementation and performance of a medicalised hotel, referral to hotel for isolation and social distancing | Data from clinical electronic register and survey conducted during stay at the hotel | N/A - not specified homeless specifically | N/A - not specified homeless specifically | Participation in survey conducted was limited and varied among questions; lack of scientific reports on medicalised hotels | Medicalised hostels are justified as providing a place to quarantine, but also allowing people to be followed up, who otherwise could not afford a safe quarantine |
| TA20 | Bharmal, 2021 (56) | London (UK),  2021 | Identified from temporary accommodation to homeless or those sleeping rough (houseless, roofless) | Outreach team working within the homeless community to address health needs: asymptomatic testing and managing of individuals with COVID-19 symptoms. | Commentary by a member of the outreach team working within the homeless community in London. | N/A | N/A | Lack of validated data on effectiveness of approach | Early data has shown that during the first wave of pandemic, London has reported fewer cases and outbreaks in homeless hostels. Outreach team benefits from working with people who have experienced homelessness in order to improve COVID-19 and other health care outcomes. |
| KS47 | Harris et al., 2020 (57) | USA, July-August 2020 | ‘Homeless’ (unclear) | Telemedicine and street outreach | Reported by patients to receive treatment for opioid use disorder. | N/A | N/A | Only two cases | Importance of awareness of the impact telemedicine and street outreach on patients with opioid use disorder has during COVID-19 outbreak. |
| HG2 (TA23) | Parkes et al., 2021 (58) | Edinburgh (UK), April-August 2020 | Beneficiaries of the centre, service staff and managers, and wider stakeholders (roofless, houseless) | Wellbeing centre - a drop-in service run by The Salvation Army, provided telephone and online communication and socially distanced in-person support over the pandemic. | Semi structured qualitative interview with clients, staff and stakeholders to assess how the Wellbeing Centre has tackled issues of substance use and harm reduction. | N/A | N/A | N/A | Telephone and online support made people feel as if they were important and valued, this also allowed staff to provide advice and psychological support when necessary. |
| ME18 | Roncero et al., 2020 (59) | Salamanca Area (Spain), March-April 2020 | Homeless ‘who could not continue to live on the streets’ (roofless) | Role of telemedicine during COVID-19 pandemic | Report of evidence that telemedicine was beneficial for the homeless population with substance abuse disorders. | N/A | N/A | N/A | Telemedicine was beneficial for the homeless population with substance abuse disorders. |
| TA40 | Aguilar et al., 2021 (60) | Salamanca (Spain), March-May 2020 | Unsheltered living in temporary accommodation (houseless) | New health care program was launched in Salamanca to avoid emergence of severe psychopathological symptoms and aggravation of the pre-existing mental illnesses in those diagnosed. | Ecological study of COVID patients over 10 weeks through professional screenings, calls and face to face visits. | N/A | N/A | N/A | Confinement due to pandemic generated maladaptive emotional responses and other behaviours, with patients under stress reverting to substance use more frequently; flexibility in providing face-to-face appointments for the most severe patients is needed, despite pandemic-related concerns. |
| ME22 | Roncero et al., 2020 (61) | Castile and Leon (Spain), April 2020 | ‘Homeless’ (unclear) | Measures applied to a drug addiction assistance network: implantation of telemedicine, restriction of daily methadone dispensing, initiation of care programs for the homeless. | Telephone survey of staff of centres was carried out to gauge opinions on impact of pandemic and repercussions on professionals and patients. | N/A | N/A | Prevention of possible increase in the development of behavioural addictions and homemade drugs. | Rapid adaptation and implementation of the network in Spain aided in preventing a possible increase in development of addictions and COVID-19 infection. |
| KS19 | Caswell et al., 2021 (62) | UK, 2020 | Temporary accommodation, homeless at home, rough sleeping, relief of duty but not accommodated by council, homeless hostel, temporary accommodation arranged by social services (houseless, roofless, insecure) | GP surgeries adapted to make it easier for PEH to register and attend appointments: remote registration systems, usage of support workers in emergency accommodation to encourage, provision of phones to clients, commissioning of homelessness healthcare service. Changes to mental health services during the pandemic included practitioners commissioned to provide flexible outreach to PEH and a lower threshold for offering mental health assessments. | Review of current policy and conducting semi- structured interviews | N/A | N/A | N/A | GP registration continues to remain an issue. Additionally, although Access to prescriptions were also provided online, it was not as comparatively beneficial in most cases. Specific focus is needed to improve current practices. |
| KS20 | Callaghan et al., 2021 (63) | Midlands and Lancashire (UK), 2020 | Homeless in temporary accommodation (TA), self-arranged TA or homeless at home (HAH), rough sleeping, living in a homeless hostel, living in TA arranged by social services (houseless, roofless, insecure) | Support for registering with a GP, telephone mental health services, outreach health care services. | Data was collected from multiple interviews and workshops with the homeless population as well as stakeholders from both public and NGOs. | N/A | N/A | Despite numerous examples of increased primary care registration, interviews and workshops with key stakeholders were not convinced this change was common. | Remote access to mental health care was negatively affecting patients due to loss of services like group therapy. There was limited access to services over the pandemic. |
| ME11 | Ferguson et al., 2021 (64) | USA, January-June 2020 | Homeless veterans as defined by outpatient stop codes (houseless, roofless, inadequate) | Overcoming access to barriers to virtual care through shift from in-person to virtual care amongst the veteran population during the early phase of COVID-19 pandemic. | Linear models to identify patient sociodemographic and clinical characteristics associated with use of phone care vs. no virtual care and use of video care vs. no video care. | N/A | N/A | Lower chances of use in homeless and older veterans to use mobile services. | Veterans with high clinical or social need proved to have a higher chance of using virtual services; however the older and homeless veterans were less likely raising concerns for access to barriers and resources during the COVID-19 pandemic. |
| KS42 | Heflin et al., 2021 (65) | Vermont, USA October/December, 2021 | Unsheltered, in temporary housing (houseless) | Medical-student led initiative to implement telehealth to the homeless population of Vermont to help with physical, mental and dental wellbeing | Descriptive paper / commentary of initiative. | N/A | N/A | N/A | 26 participants signed up, paired with 16 students, with 6 individuals unable to contact. A number of benefits have been described including maintaining long term continuity of care and building relationships virtually, however there has been difficulty reaching the homeless population over the telephone, either due to lack of access to phones, or long demanding shifts taking up a significant proportion of their time. Finally mental health problems have been hard to discuss, with many participants unwilling to discuss their issues over the phone. |
| KS28 | Benjenk et al., 2021 (66) | Washington, DC (USA), September-November 2020 | Hospitalised patients on voluntary inpatient unit: subset of group were in sheltered or unsheltered (houseless, roofless) | Social Distancing Policies, New Health and Social Service systems that have arisen, telemedicine services have been provided. | Minimally structured interviews conducted in-person by a member of the research team with 20 hospitalised adults with serious mental illness (SMI), coded using thematic analysis | N/A | N/A | Single-site study, small sample size, research team unable to use any data collection methods to triangulate the findings, information provided by study participants was not validated externally | Most participants found the new systems effective at meeting their essential needs. Unmet needs were reported, including inability to access mental health care and public benefits without identification documents, housing and/or a personal device.  8 participants described sadness in relation to social distancing policies, as they were unable to meet their family and friends or conduct their normal daily activities. an additional 6 found it difficult to access government centres or the court system, making it difficult for them to receive government welfare and receive housing.  Attitudes to telemedicine and mental health treatment during the pandemic were mixed, with some adapting well whereas others (due to lack of internet or devices) finding it much more difficult and much preferred face-to-face appointments.  A key policy implication was access to the internet and having a smartphone. Lack of these have serious implications on access to healthcare and other social services. |
| EA20 | Verity et al., 2020 (67) | Lewisham, London (UK), 2020 | Rough Sleepers, Homeless shelters (roofless, houseless) | ‘Total Triage’ (TT) where all consultations required some form of triage, and ‘Remote-by-Default’ (RbD) consulting, where clinician should consult remotely unless there is a ‘clinical exception’ | Semi-structured interviews with stakeholders working with vulnerable groups and qualitative data from forums with black and ethnic minority patients, a survey of General Practitioners exploring implementation TT and RbD), and a mystery shopper exercise reviewing access and messaging  of ten practices | N/A | N/A | N/A | There were some barriers to access for vulnerable patients including challenges navigating the new model, difficulty engaging with remote consultations and digital exclusion, and wide variation in messaging regarding changes to services and practical application of TT and RbD. |

*Alternative Housing*

| **Paper ID** | **Author** | **City (Country), study period** | **Population**  **(FEANTSA definition)** | **Policy implemented** | **How Policy Was Assessed** | **Age** | **Sex** | **Limitations** | **Main Conclusion** |
| --- | --- | --- | --- | --- | --- | --- | --- | --- | --- |
| KS44 | Coombs et al., 2021 (68) | England (UK), 2020- 2021 | Rough sleepers, accommodation where it is not possible to self-isolate e.g. communal shelters (roofless, houseless) | Everyone in scheme from government in March 2020 led to councils moving an estimated of 90% rough sleepers into accommodation | Assessed by the admission of up to 15,000 individuals being temporarily accommodate in COVID-19 compliant accommodation | N/A | N/A | However, criticisms include the constantly changing messages and advice from the government as well as the lack of support for non-UK nationals. There are also worries surrounding what support is available as we come out of the pandemic and as the economy reopens. | Scheme from government actions is effective for moving rough sleepers into COVID-19 compliant accommodation |
| KS39 | National Audit Office, 2021 (69) | England (UK), March-November 2020 | Rough sleepers, accommodation where it is not possible to self-isolate e.g. communal shelters (roofless, houseless) | “Everyone In Initiative” : providing expansive housing for rough sleepers during the pandemic | 90% (5.400) of the estimated homeless population being offered accommodation and 33,000 rehoused later in the scheme translated into only 16 deaths due to COVID with an estimated 20,000 infections and 266 deaths being avoided. | N/A | N/A | N/A | Expansive housing is effective for diminishing infection rate and deaths. |
| AR26 | Cromarty, 2021 (70) | England (UK), March 2020- January 2021 | Rough sleepers (roofless) | The Government of England launched the “Everyone In” program to protect rough sleepers during the pandemic. Around 11,263 people were provided with emergency accommodation along with further 26,167 people were moved into settled accommodations by January 2021. | The government statistics showed a significant drop to 37% in the number of rough sleepers in Fall 2020 from previous year, however, Combined Homelessness andInformation Network (CHAIN) statistics exhibited an increase of 3% in number of rough sleepers between 2019/20 to 2020/2021. | N/A | N/A |  | The initiative launched by the government reflected positive impacts on the community , thereby allowing it to support the most vulnerable population in collaboration with the local authorities, and health sector. |
| KS24 | Cookson et al., 2021 (71) | England (UK), 2020 | Rough sleeping (roofless) | Self contained accommodation (“Everyone In”) provided reducing risk of COVID-19 transmission and close work with health services. | Report of evidence that this type of emergency accommodation was beneficial for the homeless population with substance abuse disorders. | N/A | N/A | N/A | Self contained accommodation provided reducing risk of COVID-19 is beneficial for the homeless population with substance abuse disorders. |
| ME24 | Fitzpatrick et al., 2021 (72) | United Kingdom, 2020 | Rough sleepers, those at threat of eviction (roofless, insecure) | Everyone In campaign, which initiated emergency rehousing of the homeless population in hotels, holiday lets, university accommodations and other temporary accommodations | This grey literature report described the UK trends in homelessness occurring in 2021, alongside the policies enacted in response to COVID 19. Survey of local authorities, review of relevant literature, statistical analysis on relevant economic and social trends in homelessness, annual interviews with a sample of key informants from the statutory and voluntary sectors across England, and statistical modelling exercise were all used in this longitudinal study. | N/A | N/A | N/A | Over 90% of the homeless population known to councils before the pandemic were offered a place to stay, with over 33,000 people being assigned a room. In addition, the paper described policies implemented to prevent further homelessness caused by the pandemic such as the furlough scheme, legal proceedings on evictions being stopped or notice periods to be extended by at least 6 months. The survey of local authorities carried out here found that 80% of respondents found the furlough scheme to be ‘very’ or ‘somewhat’ important while 87% of councils found the eviction halting to be ‘very important’ in minimising homelessness in the area. The low levels of infection and deaths in the homeless population related to COVID reflected the success of these policies described. |
| EA41 | Marcus et al., 2020 (73) | Tshwane (South Africa), 2020 | Shelter (houseless) | Over 2000 homeless people were situated in a stadium (temporary alternative housing unit). The policies instituted included substance abuse and withdrawal support, as many of the homeless people were suffering from substance abuse. | Narrative approach along with the constructive triangulated interviews were conducted in shelters | N/A | N/A | Partiality and inconsistency in terms of time and perspective since responders narration influenced by other people response in one place | Important to communicate with people directly affected by emergencies, use methadone to wean the patients and keep control in the encampment is valuable, and imperative to include OST (opioid substitution therapy) in essential primary healthcare. |
| ME25 | Fitzpatrick et al., 2020 (74) | United Kingdom, 2020 | Rough sleepers, those at threat of eviction (roofless, insecure) | ‘Everyone In’ campaign | Early stage briefing of ‘The Homeless monitor’, which draws on authors’ analysis of policy developments and senior key informant testimony | N/A | N/A | N/A | This policy report shows that the 'Everyone In' initiative by the UK government to combat COVID-19 in homelessness was relatively successful, since over 15,000 rough sleepers which were 90% of the total homeless population were offered accommodation. Specific policies such as an enhancement on benefits as well as a halt on evictions were particularly important in preventing the increase of homelessness during the pandemic due to increases in unemployment and austerity. However, criticisms include the constantly changing messages and advice from the government as well as the lack of support for non-UK nationals. There are also worries surrounding what support is available as we come out of the pandemic and as the economy reopens. |
| JR25 | Nazroo et al., 2020 (75) | London (UK), January-July 2020 | Female rough sleepers (roofless) | Vulnerabilities in the female rough sleeper homeless population as well as their higher-risk health needs. | Preliminary data from rapid evidence review | N/A | N/A | N/A | Dramatic higher death rate in female rough sleepers  when compared to the rest of the population and shows the vulnerabilities in the homeless population as well as their higher-risk health needs. |
| HG40 (TA54) | Doctors of the World, 2020 (76) | England (UK), 2020 | People experiencing homelessness, rough sleepers (roofless) | Everyone In scheme: Funding and providing emergency accommodation for those experiencing or at risk of homelessness | Semi-structured interviews over the phone or virtually | N/A | N/A | N/A | Interviewees reported increased engagement with health services facilitated by the provision of safe and secure accommodation |
| KS17 | Whitehead et al., 2021 (77) | London, United Kingdom, July 2020-March 2021 | Anyone in danger of becoming homeless or threat of eviction from private rented sector, and rough sleepers (Insecure, inadequate, roofless) | ‘Everyone In’ initiative, and suspensions of notice by the government in order to prevent eviction | 1) Interviews with senior staff in 12 boroughs in London  2) Examination at different points in time how the suspension of evictions in the private rented sector might work its way into homelessness  3) Review of estimates of number of people assisted by ‘Everyone In’ initiative, costs to London authorities, and more generally how the initiative might be assessed | N/A | N/A | N/A | The Everyone In scheme was associated with many logistical difficulties, including a lack of space, uncertainty about how long people would need accommodation for as well as the unpredictability of many homeless people’s lives alongside the medical requirements they may have. |
| KS6 | Pennington et al., 2020 (78) | England, United Kingdom, 2020 | Temporary Accommodation (houseless) | ‘Everyone In’ campaign | Analysis of MHCLG’s statutory homelessness statistics, and telephone interviews with 21 people who lived in temporary accommodation during lockdown of 2020 | N/A | N/A | N/A | 2% (6000) increase in people living in temporary accommodation likely elucidated by onset of ‘Everyone In’ campaign. In interviews conducted, individuals living in temporary accommodation reported they didn’t feel safe and there was a lack of social distancing within their accommodation. Three people from the 20 households interview reported having to share kitchen facilities with individuals who were positive for COVID-19. In conjunction with the lack of other basic facilities e.g. laundry, washing, and the national lockdown, many people spoke of the impact of living in temporary accommodation on their mental wellbeing. |
| KS41 | Centrepoint, 2020 (79) | United Kingdom, 2020 | Rough sleeping, without access to accommodation, under threat of eviction housing (roofless, insecure) | Local authorities instructed to find emergency accommodation for all people sleeping rough or without access to accommodation, suspension of possession actions intended to protect tenants from eviction during the pandemic, change in Universal Credit allowance and Local Housing Allowance | National survey of youth homeless organisations conducted by Centrepoint in November 2020. | N/A | N/A | N/A | Regarding the Everyone In campaign, agencies associated with homelessness have felt that despite government investment e.g. 3.2 million COVID emergency funding at start of pandemic and 6 million COVID19 homeless support fund the report claims that only 22% of homeless services provide sufficient support for young people forced to sleep rough whilst 24% of the people supported by the "everyone in" scheme thought it had a positive impact for young people facing homelessness. Moreover, their survey showed that 30% of services thought that their current levels of funding were sufficient to cope with demands/challenges caused by COVID19 pandemic. |
| TA41 | Centrepoint, 2021 (80) | UK, 2021 | Youths experiencing homelessness (roofless, houseless) | Government-imposed eviction bans along with increasing universal credit by £20 a week to support those considered in the low income category. | Quantitative and Qualitative from youth homelessness charity helpline | N/A | N/A | N/A | Despite interventions by the government, Centrepoint’s helpline recorded a 33% higher number of calls in 2021/21 as compared to the year before which demonstrated that a significant amount of efforts needs to be done to address youth homelessness across the country. |
| ME23 | FEANTSA team (81) | Europe,  2021 | Rough sleepers (roofless) | Description of policies across Europe: “Everyone In” emergency accommodation was provided for the whole of the UK’s homeless and rough-sleeping population, including migrants. A mobile unit was established to test homeless people and for those who tested positive provide a designated facility under medical surveillance | Migrants are often highly represented in the numbers of rough sleepers in the big cities of Europe. This identified developments throughout the first wave of the coronavirus pandemic. | N/A | N/A | Some measures were only available for migrants with a regular status in Denmark, with individuals with uncertain migration status often not supported or guided properly | Social rights and equal access to safe accommodation for all, which has a direct impact on health outcomes affected by homelessness status, were not permanently solved during the pandemic by the measures enacted. Measures enacted for the homeless population during the pandemic should not be connected to people’s immigration status. |
| ME4 | Koh., 2020 (82) | Singapore, 2020 | Migrant workers accommodation (houseless) | The government extensively tested workers in dormitories, segregated both healthy and infected individuals, provided appropriate accommodation in which social distancing could be observed, and observed symptoms daily. | Commentary with examination of the daily numbers of COVID-19 in Singapore from March to May 2020 amongst the migrant worker population using daily reports from the Ministry of Health, Singapore | N/A | N/A | N/A | social distancing is difficult within dormitories, and that unsatisfactory housing and social overcrowding, particularly amongst this vulnerable population of migrant workers, needs to be addressed during a pandemic in order to prevent these areas becoming epicentres of the disease |
| KS21 | Healthcare Improvement Scotland, 2020 (83) | Scotland (UK), Summer 2020 | Groups mentioned: Persons living in temporary accommodation shelters, rough sleepers (houseless, roofless) | Temporary accommodation with increased engagement with health services to solve social care problems. | Qualitative investigation via semi interviews with the homeless population housed in hotels or temporary accommodation during the pandemic. | N/A | N/A | Due to the speed of the housing, some people were moved far away from informal support networks or from their GP, leading to anxious feelings. | Although better relationships were made with outreach services through this temporary accommodation, other health services and trusted relations were lost and could not grow due to isolation. |
| KS27 | Benavides et al., 2020 (84) | Dallas, Texas (USA), July 2020 | Shelters (houseless) | Provision of support for homeless people through the formation of an emergency centre at a convention centre during an outbreak at a homeless shelter | Commentary describing a case study in Dallas, Texas | N/A | N/A | N/A | Emergency housing for provision of isolation and quarantine is successful in reducing the spread of COVID, with rehousing of 164 individuals described from another shelter; however this has limited capacity as shown in quickly being fully booked, thus cannot cater to all the needs. |
| KS31 | Irwin et al., 2021 (85) | Virginia (USA), April-June 2021 | Overcrowded or congregate settings (houseless, inadequate) | Two hotels rented by the government for isolation of low-income individuals. Telephones are also purchased for the homeless population to participate in telehealth services. | Telehealth services outcomes were not described/ assessed for this policy. | N/A | N/A | Telehealth services outcomes were not described for this policy. | Isolation hotels and telephone services proved beneficial in accommodating and providing resources to vulnerable and homeless individuals. |
| ME3 | Singh et al., 2020 (86) | Chandigarh (India), 2020 | Migrants in makeshift shelter home (Houseless) | Migrants were moved to a makeshift temporary shelter home; separate rooms for families and police presence for security were arranged. | Discussion groups were conducted consisting 15-20 participants to maintain social distancing. | N/A | N/A | N/A | It is important to understand physiologic requirements and needs of migrant workers during lockdown periods. |
| ME14 | McIntyre, et al., 2021 (87) | United States America, March 24-April 19 2020. | COVID-positive (or suspected to be) hospitalised patients identified in e-health record as homeless (unclear) | Hotel arrangements made by ED social workers for placement for quarantine for SARS-CoV-2 at one specific hotel in the city funded by the state. | Conference abstract of descriptive study performed at a University tertiary-care, inner-city hospital | Average age was 49.2 years | 19/29 (65.5%) patients in total were male | N/A | After the index ED visit, 8 patients in total returned within 30 days, none of whom were admitted. Use of a hotel for homeless patients’ quarantine decreased inpatient hospital utilisation by offering an alternative to hospital admission during the SARS CoV-2 pandemic |
| HG44 | Fuchs et al., 2021 (88) | San Francisco, California (USA); March-May 2020 | Sheltered and unsheltered, unstable housing, congregate dense settings (houseless, roofless, insecure) | Hotel-based isolation and quarantine (I/Q) case system | Association with inpatient hospital capacity | 44 years (overall) | 75% male (overall) | Unable to implement a comprehensive electronic record to systematically track clinical progress and disposition of guests across referral sources. Reduced hospital length of stay may have reflected in-part improved SARS-CoV-2 test turnaround time | There is a critical need to establish an alternative housing program to contain spread of SARS-CoV-2 amongst vulnerable populations, including homeless, to prevent hospitals from becoming proxy I/Q facilities |
| JR23 | Ingle et al., 2021 (89) | Austin (USA), March 2020 | People experiencing homelessness including those in densely packed outdoor encampments or shelters  (houseless, insecure) | Examining use of isolation facilities and number of rooms needed for PEH to utilise following possible exposure to the virus | Mathematical modelling study of COVID-19 transmission | N/A | N/A | Assumption COVID-19 spreads at the same rate within the PEH community as it does in the general population. Assumption that 10% of PEH infections would be tested. | Model found that, under the worst-case scenario, 250 isolation beds were anticipated. The case study of Austin, USA showed that transmission was reduced by 50% and that between 15-130 isolation beds were used at a time, mostly by homeless individuals, reducing social contact by 75-90%. By coupling COVID-19 projections with PEH demographic information, modelling can aid in making cost-effective decisions with provision of resources. |
| AR18 | Chapman et al., 2021 (90) | USA, 2020-2021 | Sheltered (houseless) | Development of effective strategies to prevent further COVID-19 outbreaks. | Reported by microsimulation model which was based on calibration of data from homeless shelter outbreaks to find the most effective infection control practice by comparing infection control strategies to reduce COVID-19 spread amongst homeless residents. | N/A | N/A | N/A | Research found that high risk homeless shelters with high community incidence are more prone to outbreaks even with intensive infection control practices, this suggests a necessity of non-congregate housing for homeless. Furthermore, in lower-risk shelters, combined interventions (incorporation of daily symptom screening, mask wearing and repeated PCR testing) should be implemented to reduce the risk of outbreaks. |
| AR32 | Lewer et al., 2020 (6) | England (UK), February-May 2020 | People living in hostels for single homeless people, people sleeping rough, people sleeping in night shelters (roofless, houseless) | Preventative policies were implemented to protect the homeless population. A research conducted by Lewer et al. aimed to find the estimation of deaths which could be avoided among homeless people during the first wave of COVID-19. | Time Markov chain model of SARS-CoV-2 was used to find the impact of COVID-19 on 46 565 homeless individuals. A set of models was run under different scenarios by varying the infection incidence in the general population and different preventive measures available during that time. The study found that preventative measures could avoid a significant decrease in the number of cases. | N/A | N/A | Lifting preventive measures could lead to outbreaks in the homeless population settings and can further lead to higher numbers of cases and deaths. | Acknowledges the importance of preventive measures and avoiding the transmission in homeless settings including hostels and night shelters. |
| ME28 | Nande et al., 2021 (91) | Philadelphia (USA), 2020 | Evicted individuals (houseless, roofless, insecure) | Halting evictions and the effect on transmission and infection of COVID-19 | Reported by findings assessed and low eviction rate of 0.25%/month about 0.5% more of the population become infected compared to baseline scenario of no evictions. | N/A | N/A | N/A | Higher eviction rates could lead to 50-100,000 excess deaths. This shows how a small prevention of evictions can dramatically reduce the prevalence of COVID. |

*Encouragement of Personal Hygiene*

| **Paper ID** | **Author** | **City (Country), study period** | **Population**  **(FEANTSA definition)** | **Policy implemented** | **How Policy Was Assessed** | **Age** | **Sex** | **Limitations** | **Main Conclusion** |
| --- | --- | --- | --- | --- | --- | --- | --- | --- | --- |
| ME9 | Foster-Bey et al., 2021 (92) | Detroit, USA, Spring-Summer 2020 | Sheltered, Encampment (houseless) | Creation of field hand washing stations, which were located at soup kitchens and homeless encampments, with refilling and sanitising of the stations twice weekly. | Informal testimonials from homeless individuals who used the station | N/A | N/A | N/A | Hand hygiene stations require relationship building and engagement with the homeless community to be effective, but has become integral to health maintenance for some individuals experiencing homelessness. |
| EA39 | Montgomery et al., 2021 (93) | Atlanta, Georgia (USA), May-August 2020 | Sheltered and unsheltered homelessness (houseless, roofless) | Hand hygiene practices included existing shelter rooms and restrooms in public facilities, non-profit organisations and individuals distributing hygiene supplies to unsheltered and sheltered participants | In-depth semi-structured interviews conducted on homeless individuals, conducted in person by homelessness outreach staff provided with qualitative interview training, | Median year for unsheltered: 50 years. Median year for sheltered: 49 years | Unsheltered: 12 male, 11 female. Sheltered: 14 male, 14 female | Small sample size, interviews with people experiencing unsheltered homelessness were conducted earlier than interviews with people living in shelter | The most significant barrier was lack of access to facilities and services following the closure of public facilities and services during the pandemic. This led to increased use of hazardous water sources by the unsheltered population. Most participants viewed hand hygiene as common sense and preferred access to supplies over education. Installing portable hand washing stations or providing supplies in small, portable containers on regular basis can help address these access barriers |
| KS43 | Davies et al., 2020 (94) | Pennsylvania, USA, 2020 | Youth shelter (houseless) | Youths in the shelter were encouraged to undertake to wear masks, through education, encouraging decoration of masks, and provision of cloth masks over surgical masks. | Monitoring number of youths wearing masks, qualitative commentary of interventions implemented. | N/A | N/A | N/A | Allowing personalised cloth masks brought mask wearing to the majority. The shelter now reports ¾ of residents wearing masks, 6 weeks after the mask wearing initiative was started. |
| SM4 | Steer et al., 2021 (95) | Calgary, Alberta, May-June 2020 | Emergency shelter, supportive housing site, encampment team (rough sleepers), shelters, unstable housing, urban individuals experiencing homelessness, supportive housing (houseless, roofless, insecure) | Provided disposable paper cups to service providers for distribution to clients | Service providers tracked number of cups distributed, and staff and peers who distributed the cups were interviewed to assess effectiveness | N/A | N/A | Correlating epidemiologic trends to the intervention was limited by an overall Calgary-wide decrease in COVID-19 cases and confounding with the various outbreak containment efforts simultaneously implemented. | Disposable cups are a rapidly implementable, low-cost harm reduction tool to empower people experiencing homelessness to reduce the risk of COVID-19 transmission due to drink sharing. It should be implemented as part of a larger harm reduction and community education strategy |
| EA42 | O’ Carroll et al., 2020 (96) | Dublin, (Ireland), 2020 | Sheltered and unsheltered (houseless, roofless) | Opioid-substitution therapy: methadone and benzodiazepine were offered to homeless patients suffering from substance abuse. Implementation of protocols to identify and isolate homeless clients with symptoms | Descriptive paper of response to the COVID-19 pandemic in the Dublin Homeless Community | N/A | N/A | Difficult to disentangle the individual impacts of housing, harm reduction and health service provision interventions on infection and mortality rates. Paper does not include any feedback from the population in question | Opioid-substitution therapy helped reduce covid infections by reducing contact and contamination as prevented sharing of needles. Further housing measures undertaken by the city drastically reduced rate of infections, with the rate amongst homeless people at 2% compared to 1% for Dublin, and only one covid-related death which was much lower than expected. |

*Inter-organisational Communication*

| **Paper ID** | **Author** | **City (Country), study period** | **Population**  **(FEANTSA definition)** | **Policy implemented** | **How Policy Was Assessed** | **Age** | **Sex** | **Limitations** | **Main Conclusion** |
| --- | --- | --- | --- | --- | --- | --- | --- | --- | --- |
| KS12 | Honorato et al., 2020 (98) | Brazil, December 2019-March 2020 | Sheltered, recovery institutions, Rough sleepers (houseless, roofless) | Brazilian government did not implement any measures for the homeless population. Policies implementing, including adaptation and building new temporary shelters, education on how to tackle disease and distribution of food and hygiene related items, were implemented by individuals and NGOs working with homeless population | Data was collected from interviews with people involved in NGOs and charities which help with homeless people. | N/A | N/A | Disconnect between government and individual measures. | The effects of the measures were not specifically addressed due to a disconnect between government advice and individual measures to help prevent COVID in the homeless population, the paper describes high burnout and tolls on workers physically and on their mental health. Paper also stresses the importance of strong leadership to tackle the problem. |
| TA39 | Jain et al., 2021 (99) | USA, February-March 2021 | Sheltered, Emergency Shelters (houseless, roofless) | Prioritisation of vaccination among people living in congregate settings against federal guidance | Rapid review analysing several states in the U.S. | N/A | N/A | N/A | It is important to prioritise vaccines amongst people living in congregate settings due to higher risk of disease; socially vulnerable groups were prioritised by states despite not being included in federal guidance. Prioritisation of groups for vaccination has been highly variable. |
| AR16 | Yu et al., 2020 (100) | England (UK), November 2020 | ‘Homeless’ group, migrants, asylum seekers, refugees (unclear) | Assess quality of local outbreak control plans (LOCP): rapid response to cases, advice on getting tested, preventing positive workers from going to work, and support for homeless people to self-isolate. | Statistical analysis of LOCPs, assessment based on Department of Health and Social Care guidelines framework and qualitative analysis | N/A | N/A | Small sample size in each local authority group, variation in definitions across high-risk settings, only examined specific high-risk settings | Homeless populations were well-represented in policies in the UK; this could be due to more funding from the UK government potentially increasing focus on this group. |
| AR21 | Alavi et al., 2021 (97) | Iran, March-April 2020 | Sheltered, unsheltered (houseless, roofless) | The Working Group initiated the distribution of resources in four provinces in Iran among people who use drugs. Also provided education sessions and carried out routine checks to diagnose positive COVID-19 cases. A finalised protocol was published by the government to accommodate the vulnerable population by considering the medical, housing, transportation and harm reduction needs recommended aimed to provide continued support. | Qualitative commentary | N/A | N/A | Continuous monitoring of the outcomes to establish more effective health policies remains the need for the Iran government. | NGO preventative measures to arrange COVID-19 response amongst the vulnerable population proved beneficial regarding harm reduction needs in Iran. |

Title: Study characteristics of studies looking at non-pharmaceutical intervention
